# Bacterial resistance across habitats: from German schools to the International Space Station

**DOI:** 10.3389/fmicb.2026.1849378

**Published:** 2026-06-18

**Authors:** Carolin Luisa Krämer, Franca Arndt, Alessa Lalinka Boschert, Bernd Walkenfort, Stefan Leuko, Mike Hasenberg, Kristina Beblo-Vranesevic, Katharina Döscher-Siems

**Affiliations:** 1 Department of Natural Sciences, University of Applied Sciences Bonn-Rhein-Sieg, Rheinbach, Germany; 2 Department of Applied Aerospace Biology, Institute for Aerospace Medicine, Cologne, Germany; 3 Institute for Medical Microbiology, Immunology and Hygiene, University Hospital of Cologne, Cologne, Germany; 4 MVZ Laboratory Dr. Limbach & Colleagues eGbR, Heidelberg, Germany; 5 Imaging Center Essen, Electron Microscopy Unit, Faculty of Medicine, University of Duisburg-Essen, Essen, Germany

**Keywords:** antibiotic resistance, bacterial resistance, habitats, radiation, space microbiology

## Abstract

Environmental factors play a crucial role in the emergence, persistence, and spread of bacterial resistance. The habitat of microorganisms can define their stress resistance profile. Through interaction with other microorganisms, the exchange of microbial resistance traits can be facilitated, thereby enhancing their overall resilience. Microorganisms exposed to space-related stressors, such as microgravity, increased levels of ionizing radiation, and desiccation, face distinctive survival challenges. These stressors can induce cellular adaptations that can affect microbial resistance. Moreover, confinement and cleaning routine can further drive the evolution of more resistant strains that could influence microbial communities in spacecraft habitats. Hence, assessing the resistance to multiple stressors is critical for understanding how different habitats shape resistance profiles and for developing microbial control strategies that may help ensure the safety of space missions. This is particularly important as humanity plans for prolonged space travel and habitation on extraterrestrial surfaces. This study explores the stress resistance of bacterial isolates collected from the International Space Station and German schools in the context of the spaceflight experiment “Touching Surfaces,” which tested antimicrobial surfaces on the ISS and on Earth. The isolates were tested for their antibiotic resistance revealing no detectable increase in antibiotic resistance under the conditions tested in isolates from space or schools. Additionally, isolates of the skin commensal *Micrococcus luteus* and the opportunistic pathogens *Staphylococcus haemolyticus*, *Staphylococcus epidermidis* and a methicillin-resistant *Staphylococcus aureus* (MRSA) were tested for their tolerance to key environmental stressors relevant to spaceflight. Stress resistance assays included desiccation, X-ray irradiation, and hydrogen peroxide treatment. Overall, space isolates did not show an increased resistance to desiccation or, X-ray irradiation. However, both space and school isolates of *Staphylococcus* spp. recovered from surfaces showed increased survival after treatment with hydrogen peroxide compared to their respective type strains. Additionally, early-stage biofilm formation was significantly higher for space and school isolates of *Micrococcus* sp. compared to the type strain *M. luteus*.

## 1 Introduction

Microbial resistance against various conditions in the inanimate environment such as increased tolerance to desiccation and antimicrobial surfaces, is a critical concern across diverse environments, including built, clinical, and extraterrestrial habitats, since selective pressure and microbial adaptation allow resistant strains to persist and spread in any ecosystem where host-microorganism interactions occur. Bacterial resistance can be divided into intrinsic and acquired resistance. Intrinsic resistance is inherent to the organism and can involve cellular envelope permeability, constitutive efflux, and low-affinity drug targets (Russell, 1995; Vila et al., 2007; Hollenbeck and Rice, 2012; Cox and Wright, 2013). Acquired resistance is driven by mutation or external DNA uptake, and accelerated by antibiotic misuse in medicine, agriculture, and veterinary practices [reviewed in more detail in Larsson and Flach (2022), Manyi-Loh et al. (2018), and Chaw et al. (2018)]. Antimicrobial resistance (AMR) undermines treatment efficacy, increases disease burden and healthcare costs, and poses a growing global threat as once-treatable infections become increasingly difficult to manage [as reviewed elsewhere, e.g., in Ahmed et al. (2024), Salam et al. (2023)]. Additionally, environmental stressors have been shown to drive evolution towards increased antibiotic resistance through the spread and accumulation of AMR (Coque et al., 2023) and resistance to other stressors has been identified to increase antibiotic resistance (as reviewed in Murray et al. (2024)). For example, the usage of copper and zinc as micronutrients in feedstock in combination with supplemented antibiotics can later result in co-selection to metals and increased antibiotic resistance (Wales and Davies, 2015; Imran et al., 2019; Yazdankhah et al., 2014). Exposure to inorganic antimicrobials, such as metals (e.g., copper and zinc), can affect antibiotic resistance in multiple species, including the clinically relevant methicillin-resistant *Staphylococcus aureus* (MRSA), and might thus accelerate antibiotic resistance (Romero et al., 2017; Cavaco et al., 2011).

However, environmental factors, including anthropogenic stressors, can not only increase antibiotic resistance rates, but also promote co- and cross resistances. While there are different terminologies which are used, in this publication it refers to the following: The evolution of co-resistance occurs when bacteria acquire and maintain multiple resistance determinants, often linked on mobile genetic elements. Cross-resistance or pleiotropic resistance, on the other hand, involves a single genetic mechanism such as an efflux pump or membrane-permeability [as reviewed and explained in greater detail in Cantón and Ruiz-Garbajosa (2011)], resulting in resistance to multiple stressors. Both co- and cross-resistance are facilitated by horizontal gene transfer and enable simultaneous resistance to different classes of antimicrobials, thereby enhancing their survival under diverse selective pressures (Sheikh et al., 2025). Isolates of *Pseudomonas aeruginosa* from different habitats, including wastewater treatment plants as well as clinical and veterinary settings, showed simultaneous resistance to multiple antibiotics and disinfectants, and, hence, demonstrated antibiotic-biocide efflux pump driven cross-resistance, confirming laboratory studies investigating cross-resistances (Amsalu et al., 2020).

One important anthropogenic factor is the built environment. Built environments can shape microbial communities by imposing environmental constraints, and thereby may influence habitat-associated resistance patterns. Due to their characteristics of being highly-maintained, the microbiome of surfaces of confined environments is distinctly different (Mahnert et al., 2019; Lang et al., 2017). The factors that are considered to be of significance in this context besides confinement, resulting in decreased microbial diversity, include increased cleaning protocols, desiccation, nutrient limitation, and the main microbial input from the human microbiome (Mahnert et al., 2019). Hence, the above-described influence might be even more magnified in confined environments, such as intensive care units in hospitals, as well as in the context of spaceflight, assembly cleanrooms, and spacecrafts themselves.

One extreme environment to study the effect of confinement and severe external stressors is the International Space Station (ISS). While higher diversity of resistance genes correlated with confinement and loss of microbial diversity, factors relevant for human health were not increased in microbial communities on the ISS (Mora et al., 2019; Mahnert et al., 2019). Additionally, studies have shown that, while the microbiome of the ISS is unique and human-associated, it is similar to that of terrestrial confined environments (Mora et al., 2016a). However, multi-omics analyses of the ISS microbiome revealed distinct metabolomic and genomic profiles compared to those of other terrestrial environments (Salido et al., 2025). Beyond confinement like in intensive care units and clean rooms, the ISS represents an extreme habitat with additional environmental stressors such as increased radiation and microgravity. This imposes a unique set of selective pressures towards microorganisms (Horneck et al., 2010). Comparative genome analysis revealed that microorganisms on the ISS might have adapted to the unique conditions and thus became more resistant to space-related stressors (Szydlowski et al., 2024). Moreover, studies showed that tendencies towards surface attachment and biofilm formation were enhanced in ISS isolates and biofilm formation of *P. aeruginosa* increased under spaceflight conditions (Mauclaire and Egli, 2010; Kim et al., 2013; Mora et al., 2016b). Persistence and adaptation of microorganisms do not only include resistance to antibiotics but also to disinfectants, radiation, desiccation, and metal surfaces. Therefore, studying the unique resistance profiles of isolates from different habitats, such as the ISS, is crucial in order to better be able to classify potential hazards arising from adaptation to spaceflight. This may aid not only in protecting crew health, but also the material integrity of the respective spacecraft.

An environment completely contrary to the controlled confined environments of cleanrooms and spacecrafts is schools. This environment is oftentimes overlooked in research, but crucial to our society. Schools represent non-confined, densely populated environments with high contact frequency. Studies have demonstrated that the microbiome of school environments primarily consists of human-associated bacteria, and its composition depends on patterns of use and occupancy frequency (Lee et al., 2021; Feng et al., 2021; Meadow et al., 2014). The most predominant genera identified include *Enhydrobacter*, *Micrococcus*, and *Staphylococcus* (Lee et al., 2021). Additionally, cleaning frequency in schools is sometimes limited, and many objects are shared among a great number of students. In the midst of the global COVID-19 pandemic, schools were among the first infrastructure to be shut down. One study investigating the air microbiome of different classrooms found that the environment of the school (i.e., urban or rural) influences the school microbiome, amongst other factors such as disinfection routine (Yang et al., 2022). Furthermore, some studies have associated the distinct classroom microbiome, as compared to the home microbiome, with increased asthma symptoms, underscoring the need for further investigation (Lai et al., 2018). The interplay between microorganisms, environmental characteristics, and asthma is complex (Fu et al., 2020). Currently, classroom-level microbiome data are limited, but research in school environments may enhance understanding of indoor habitats and their impact on human well-being.

Built environments act as selective filters that shape microbial communities through environmental constraints and influences on microbial dispersal and survival. The ISS, as a model of an extreme built habitat, illustrates how these pressures can drive microbial adaptation and resistance. Schools, on the other hand, represent a crucial, terrestrial everyday habitat, with high human microbial input. Despite increasing recognition that built environments shape microbial resistance, comparative studies across extreme and everyday habitats remain limited. Hence, in this study we aimed at investigating whether the different isolation environments in everyday-terrestrial and extreme-space habitats influence stress tolerance. This study was focused on bacterial isolates collected within the “Touching Surfaces” experiment from high-touch metal surfaces from the ISS and German schools (Krämer et al., 2025).

Building from this, the objective of the present study was to evaluate whether these distinct environments yield bacterial isolates with differing stress resistance profiles. We hypothesize that isolates from confined and extreme environments (ISS) exhibit enhanced tolerance to multiple stressors compared to isolates from high-contact terrestrial environments (schools).

## 2 Materials and methods

### 2.1 Isolation of bacteria and used strains

Bacteria were isolated within the frame of the “Touching Surfaces” spaceflight experiment. Within “Touching Surfaces” antimicrobial surfaces were implemented in so-called Touch Arrays, which were then installed on the ISS and in German schools, where they were frequently touched by astronauts and students, respectively (Krämer et al., 2025). Afterwards, surfaces, which included stainless steel, copper, and brass with three different topographies each (polished, 3 μm, 800 nm), were swabbed for the isolation of microorganisms with a focus on human-associated and clinically relevant isolates. Swabs were added to Brain Heart Infusion Broth (Merck Millipore, Germany) and incubated at 37 °C, 200 rpm for 48 h. The isolation and identification procedure as well as an overview of all the different species which were isolated is given in Krämer et al. (2025). Within Touching Surfaces, a total of 33 isolates were recovered from Touch Arrays placed in schools, while 23 isolates were obtained from Touch Arrays on the ISS. Some isolates of one surface belonged to the same species and may represent replicates of a single strain. *Bacillus*, *Staphylococcus*, and *Micrococcus* were the most prevalent genera; therefore, first assessment of resistance potential through antibiotic resistance testing focused on isolates from these genera.

A more detailed overview of all isolates used in this study is given in Table 1.

**Table 1:** Used isolates and type strains in this study in respect to their isolation origin.

Species	Strain	Abbreviation	Isolation origin	Source
ISS strains				
*Bacillus cereus*	W2_S1 K3	–	Steel, polished	Krämer et al. (2025)
*Bacillus cereus*	W2_S2b K1	–	Steel, 800 nm	
*Bacillus cereus*	W3_S1 K1	–	Steel, polished	
*Bacillus subtilis*	W3_S1 K9	–	Steel, polished	
*Bacillus subtilis*	W2_B1 K4	–	Brass, polished	
*Staphylococcus haemolyticus*	W2_B1 K1	*S. haemolyticus* ISS	Brass, polished	
*Micrococcus luteus*	W4_Al K1	*M. luteus* ISS	Aluminum case	
School strains				
*Bacillus subtilis*	A6_S1 K1	–	Steel, polished	Krämer et al. (2025)
*Bacillus subtilis*	A6_C2a K1	–	Copper, 3 μm	
*Staphylococcus epidermidis*	A2_S2b K1	*S. epidermidis* School	Steel, 800 nm	
*Micrococcus luteus*	A4_Al K1	*M. luteus* School 1	Aluminum case	
*Micrococcus luteus*	A1_Al K4	*M. luteus* School 2	Aluminum case	
*Micrococcus luteus*	A4_C1 K1	*M. luteus* School 3	Copper, polished	
*Staphylococcus aureus*	1.1_7	*S. aureus* School	Contact plate (TSA)	
Type strains				
*Staphylococcus aureus*	DSM20231^T^	*S. aureus* type	Human pleural fluid	Schleifer and Kocur (1973) and Meyer and Schleifer (1978)
*Staphylococcus epidermidis*	DSM20044^T^	*S. epidermidis* type	Nose	Hugh and Ellis (1968)
*Staphylococcus haemolyticus*	DSM20263^T^	*S. haemolyticus* type	Human skin	Kloos and Schleifer (1975)
*Micrococcus luteus*	DSM20030^T^	*M. luteus* type	Unknown	Schleifer et al. (1972), emend. Nouioui et al. (2018)

The type strains were used as a reference. Most isolates from ISS and schools were recovered from Touch Arrays. The *S. aureus* school strain was recovered from a tryptic soy agar contact plate, which was previously touched by students. All type strains were obtained from the German Collection of Microorganisms and Cell Cultures (DSMZ), Braunschweig, Germany. ^T^ is standing for the type strain.

For easier reading in the following *M. luteus* W4_Al K1 will be referred to as *M. luteus* ISS, *M. luteus* A4_Al K1 as *M. luteus* School 1, *M. luteus* A1_Al K4 as *M. luteus* School 2, and *M. luteus* A4_C1 K1 as *M. luteus* School 3.

*Staphylococcus* spp. will be referred to with their respective species and isolation origin (ISS/School). Type strains will be referred to as Type along with their respective species.

### 2.2 Antibiotic susceptibility testing

Antibiotic susceptibility testing of isolates was performed following clinical standard practice according to the EUCAST (European Committee on Antimicrobial Susceptibility Testing) breakpoint recommendations [Clinical breakpoints (v 15.0)] (EUCAST, 2025). For antibiotic susceptibility testing of *Bacillus* spp. and *Staphylococcus* spp., the respective guidelines for their genus were used. Since there are no breakpoints for *M. luteus* specified, for antibiotic resistance testing of *M. luteus* pharmacokinetic/ pharmacodynamic (PK/PD) breakpoints from the EUCAST guide on “When there are no breakpoints 2024-02-29” were used (v15.0) (EUCAST, 2024). Due to currently lacking species-defined clinical breakpoints for *M. luteus*, β-lactams as a readily available therapeutic options, as well as further potential treatment options vancomycin and linezolid were chosen for testing. To determine the minimal inhibitory concentration (MIC) for *Staphylococcus* spp. routine diagnostic was performed using a Vitek® (Biomerieux, France) and microbroth dilution assay plates (Supplementary Table 1). For testing of *M. luteus* and *Bacillus* spp., MIC test stripes (e-tests) were used (Supplementary Table 2).

### 2.3 Preparation of cell suspensions

Bacterial cell suspensions were used for stress exposure assays, preparation for scanning electron microscopy (SEM), and crystal violet assays. For the preparation of bacterial cell suspensions, 20 mL of tryptic soy yeast broth (TSYB: 17 g/L casein peptone, 3 g/L soy peptone, 5 g/L NaCl, 3 g/L yeast extract, 2.5 g/L K_2_HPO_4_, 2.5 g/L glucose, pH 7.3) were inoculated with the respective strain and incubated at 37 °C and 150 rpm for 18 h. After incubation, cells were centrifuged at 4000 x *g* for 5 min. Subsequently, the cell pellet was washed with phosphate buffered saline (PBS: 7 g/L Na_2_HPO_4_, 3 g/L KH_2_PO_4_, 4 g/L NaCl, pH 7.5) and afterwards resuspended in 10 mL PBS. The number of cells in the bacterial suspension was adjusted by measuring the optical density at 600 nm (OD_600nm_) using a microplate reader (BioTek ELX808, Agilent, USA).

### 2.4 Stress exposure assays

For stress exposure assays, cell suspensions were prepared as described in section 2.3. The cell number was adjusted to an optical density at 600 nm (OD_600nm_) of 0.3 corresponding to 10^8^ CFU/mL for *Staphylococcus* spp. strains and 10^7^ CFU/mL for *M. luteus* strains if not indicated otherwise. To determine the survival after exposure, 10-fold dilution series were prepared in PBS, and each step was plated on 1/8 of a tryptic soy yeast agar plate (TSYA: 17 g/L casein peptone, 3 g/L soy peptone, 5 g/L NaCl, 3 g/L yeast extract, 2.5 g/L K_2_HPO_4_, 2.5 g/L glucose, 15 g/L bacteriological agar, and pH 7.3) to determine colony forming units per mL (CFU/mL). Survival fractions were determined by dividing the cell count after exposure (N) by the initial cell count (N_0_). For all stress exposure assays three independent experiments were conducted in biological triplicates each.

#### 2.4.1 Desiccation assay

To investigate the survival of the selected isolates after desiccation, bacterial cell suspensions were prepared as described above and the OD_600nm_ was adjusted to 3. In total six 96-well plates were prepared in the following way: Per strain, three wells were filled with 20 μL of the prepared bacterial cell suspension including control wells with 20 μL PBS each. For the initial cell count, 180 μL TSYB were added directly before drying out. For all other timepoints, the 96 well-plates were left to dry under a laminar flow bench overnight. After 1, 7, 14, 21, 28, and 56 days, respectively, 200 μL TSYB were added to each well, and samples were resuspended. Subsequent to this, survival was determined as previously outlined by CFU determination.

#### 2.4.2 X-ray irradiation

X-ray treatment was performed as described in Cortesão et al. (2020). For irradiation, 100 μL of the prepared cell suspension (as described in section 2.3) were transferred into PCR tubes. Samples were irradiated with X-rays (200 kV, 15 mA) at cumulative doses of 50, 100, 250, and 500 Gy using a closed X-ray system (RS 225, Gulmay, United Kingdom). The dose rate was determined using a TM30013 ionization chamber connected to a UNIDOS^webline^ dosimeter (PTW, Germany) with an average dose rate of 15.7 Gy/min ± 2.4 Gy/min. To exclude absorbance of radiation by the used PCR tubes, a PCR tube was cut open and placed on top of the dosimeter during determination of the dose rate. To determine the initial cell count at 0 Gy, untreated cell suspension was used. For X-ray irradiation the lethal dose for 90% (LD_90_) of the cells was calculated via linear regression of the survival fraction data.

#### 2.4.3 Oxidative stress assay using hydrogen peroxide

For assessment of survival after exposure to hydrogen peroxide, cell suspensions were prepared as described earlier. In a 24-well plate, 900 μL cell suspension were added to one well per replicate. To reach final H_2_O_2_ concentrations of 1.5 and 3% in the cell suspension, 100 μL of a 15 and 30% H_2_O_2_ solution were added, respectively. To stop the H_2_O_2_ exposure, 30 μL were taken out of each well after 15 min and 30 min, respectively, and added to 270 μL of a 1 mg/mL catalase solution dissolved in PBS. To determine the initial cell count, 30 μL of untreated cell suspension were added to 270 μL of 1 mg/mL catalase solution in PBS.

### 2.5 Scanning electron microscopy

For scanning electron microscopy (SEM) one sterile glass disc with a 12 mm diameter each was put into wells of a 24-well plate. Glass discs were then sterilized with UV-C irradiation for 60 min. For inoculation of the glass discs, 600 μL of previously prepared bacterial suspension in TSYB with an OD_600nm_ of 0.3 were added to each well. The plate was then incubated at 37 °C for 24 h. To fix the cells, medium was carefully replaced with fixative [4% formaldehyde, 2.5% glutaraldehyde in 0.1 M PHEM buffer (60 mM PIPES, 25 mM HEPES, 10 mM EGTA, 2 mM MgCl_2_)] and incubated at 4 °C for 24 h. For long-term storage, 1x fixative was replaced with a 1% formaldehyde solution in 0.1 M PHEM buffer.

Imaging was conducted using a Zeiss Crossbeam 540 at 3 kV high tension and a beam current of 1 nA. Images were acquired with a pixel size of 5 nm using Zeiss Smart SEM software (Carl Zeiss Microscopy GmbH, Germany).

### 2.6 Crystal violet biofilm formation assay

To investigate biofilm formation, the semiquantitative crystal violet assay was performed based on Stepanović et al. (2000) and subsequently modified. Bacterial cell suspensions were prepared as described before and the OD_600nm_ was adjusted to 0.3 in TSYB. All strains were incubated statically in 300 μL in a 96-well plate for 24 h at 37 °C. After incubation, the supernatant was carefully discarded, and the potential biofilm was washed twice with 300 μL PBS each. Afterwards, the plate was left to dry for 10 min under the laminar flow hood. For dyeing of the biofilm, 200 μL of 0.5% crystal violet (Merck, Germany) solution in 25% methanol was added. For incubation the plate was wrapped in aluminum foil and incubated for 30 min at room temperature. The supernatant was discarded and cells were washed twice using 300 μL ddH_2_O each. To dissolve the crystal violet stain from the biofilm, 300 μL 95% ethanol were added each and the plate was placed on a shaker for 5 min. The dissolved stain was measured using the absorbance at 570 nm using a microplate reader (Infinite M200 PRO, Tecan, Switzerland). The crystal violet biofilm assay was performed in three independent experiments with three biological replicates each. Absorbance at 570 nm was measured in 16 technical replicates per well and the mean absorbance was used for calculation.

### 2.7 DNA extraction and whole genome sequencing

For genome analysis, bacteria were grown in TSYB to an OD_600nm_ of approximately 0.7 at 37 °C and 180 rpm. Subsequently, 10 mL of cells were pelleted, and washed twice with PBS. The resulting cell pellet was resuspended in 1 mL Nucleic Acid Prevention (NAP) buffer (Microsynth AG, Switzerland). Samples were shipped to Microsynth AG, Switzerland, where DNA extraction and subsequent Oxford Nanopore Technologies sequencing were performed according to the provided protocol. Written approval was obtained from Microsynth AG. Bacterial pellets were resuspended in 400 μL TE buffer (100 mM Tris, pH 8, 10 mM EDTA) containing 15 mg/mL lysozyme (Merck, Germany) and incubated overnight at 37 °C. Proteinase K (Genaxxon, Germany, final concentration 2 mg/mL), RNase A (final concentration 0.5 mg/mL), and SDS (final concentration 0.5%) were then added, followed by incubation for 30 min at 56 °C. DNA was extracted using the Quick-DNA™ HMW MagBead Kit (ZymoResearch. Germany) according to the manufacturer’s instructions on a KingFisher Flex Instrument (ThermoFisher, USA). DNA quantification was performed using the Pico488 dsDNA assay (Lumiprobe, Germany). Library preparation was conducted using the Rapid Barcoding Kit 96 (SQK-RBK114.96; ONT) following the manufacturer’s instructions. The ONT Rapid DNA libraries were sequenced on a PromethION apparatus with a FLO-PRO114M flow cell. Raw ONT sequencing data were base-called, demultiplexed, and adaptor-trimmed using the software dorado (ONT). Long-reads in fastq format were quality checked and filtered, discarding any reads with an average quality below Q10 and a read length below 1,000 bp. The remaining long-reads were assembled into contigs using the software flye (Kolmogorov et al., 2019) and subsequently polished. Average nucleotide identity was determined using the Prokaryotic Genomes Annotation Pipeline (Ciufo et al., 2018). Annotated sequences were generated using the Prokaryotic Genome Annotation Pipeline (Li et al., 2021).

Comparative analysis was performed using EDGAR 3.0 (Dieckmann et al., 2021). Antibiotic resistance and virulence genes were retrieved via Galaxy (25.1.1.dev0) using ABRicate (version 1.0.1) (Seemann, 2016) with different databases including resfinder (Florensa et al., 2022), VFDB (virulence factor database) (Zhou et al., 2024; Chen et al., 2005), NCBI Bacterial Antimicrobial Resistance Reference Gene Database and megares (Bonin et al., 2022). Parameters of ABRicate were set to default with minimum DNA coverage and minimum DNA identity each set to 80%.

### 2.8 Statistical analysis

Statistical analysis to determine significance of stress exposure tests of *M. luteus* strains was performed using Sigmaplot 14.5 (Inpixon GmbH, Germany). For determination of statistical significance between survival of *M. luteus* strains, a one-way analysis of variance (ANOVA) was performed. When the Shapiro–Wilk test for normality and the Brown-Forsythe test for equal variance was passed, and the differences in the mean values were greater than expected by chance, the Holm-Sidak method was performed as a pairwise multiple comparison procedure. When there was no equal variance (*p* < 0.05), Tukey’s test was performed as a multiple comparison procedure. When the Shapiro Wilk test for normality failed (*p* < 0.05) a Kruskal-Wallis one-way ANOVA on ranks was performed.

For statistical analysis of *Staphylococcus* spp., a student’s *t*-test was performed to determine significant differences between the isolate and its respective type strain. When normal distribution was not assumed, a Man-Whitney-U test was performed.

## 3 Results

### 3.1 Antibiotic resistance of isolates from different habitats

An overview of the bacterial strains, which were used within this study, is given in Table 1 in respect to their isolation origin. School and space isolates which were retrieved within the frame of the spaceflight experiment “Touching Surfaces” were tested according to the EUCAST breakpoint guidelines, following standard clinical diagnostic procedures. Antibiotic resistance of *Bacillus* species is given in Table 2, for *Staphylococcus* species in Table 3, and for *M. luteus* in Table 4. Following the EUCAST guidelines, S is susceptible at standard dosing regimen, R is resistant when the likelihood is high of therapeutic failure, and I is defined as susceptible at increased exposure, meaning that therapeutic success is more likely when dose is increased (EUCAST, 2026).

**Table 2:** Antibiotic resistance of *Bacillus* strains.

Species	Strain	Origin	Vancomycin	Linezolid	Clindamycin	Ciprofloxacin	Meropenem
*Bacillus subtilis*	A6_S1 K1	School	0.38 μg/mL	2 μg/mL	1.5 μg/mL	0.94 μg/mL	0.94 μg/mL
*Bacillus subtilis*	A6_C2a K1	School	0.5 μg/mL	2 μg/mL	1.5 μg/mL	0.94 μg/mL	0.64 μg/mL
*Bacillus subtilis*	W3_S1 K9	ISS	0.19 μg/mL	1 μg/mL	0.5 μg/mL	0.94 μg/mL	0.125 μg/mL
*Bacillus subtilis*	W2_B1 K4	ISS	0.38 μg/mL	1.5 μg/mL	0.75 μg/mL	0.64 μg/mL	0.125 μg/mL
*Bacillus cereus*	W2_S1 K3	ISS	0.5 μg/mL	0.38 μg/mL	1.5 μg/mL	0.125 μg/mL	0.32 μg/mL
*Bacillus cereus*	W2_B2a K2	ISS	0.38 μg/mL	1 μg/mL	0.75 μg/mL	0.125 μg/mL	0.64 μg/mL
*Bacillus cereus*	W3_S1 K1	ISS	0.5 μg/mL	2 μg/mL	0.38 μg/mL	0.75 μg/mL	0.38 μg/mL

MIC values were determined using *e*-tests. The values which are above the resistance threshold for *Bacillus* spp. determined by EUCAST are marked in grey.

**Table 3:** Antibiotic resistance of *Staphylococcus* strains.

Screening	*S. epidermidis* School	*S. haemolyticus* ISS	*S. aureus* School
Cefoxitin screening	Negative	Negative	Positive
Oxacillin	S (≤0.25 μg/mL)	S (≤0.25 μg/mL)	R (≥ 4 μg/mL)
Flucloxacillin	S	S	–
Ampicillin	S	S	R
Cefazolin	S	S	R
Cefuroxime	S	S	R
Cefpodoxime	S	S	R
Imipenem	S	S	R
Meropenem	S	S	R
Piperacillin/Tazobactam	S	S	R
Gentamycin	S (≤0.5 μg/mL)	S (≤0.5 μg/mL)	R (≥ 16 μg/mL)
Levofloxacin	I (≤0.12 μg/mL)	I (≤0.12 μg/mL)	I (0,25 μg/mL)
Ciprofloxacin	I	I	I
Clindamycin	S (≤0.12 μg/mL)	S (≤0.12 μg/mL)	R (≥ 4 μg/mL)
Erythromycin	S (≤0.25 μg/mL)	R (≥8 μg/mL)	R (≥ 8 μg/mL)
Linezolid	S (1 μg/mL)	S (2 μg/mL)	S (2 μg/mL)
Daptomycin	S (0.5 μg/mL)	S (≤0.12 μg/mL)	S (0.25 μg/mL)
Vancomycin	S (1 μg/mL)	S (≤0,5 μg/mL)	S (1 μg/mL)
Tetracycline	R (2 μg/mL)	S (≤1 μg/mL)	R (2 μg/mL)
Tigecycline	S (≤0.12 μg/mL)	S (≤0.12 μg/mL)	S (≤ 0.12 μg/mL)
Fusidic acid	S (≤0.5 μg/mL)	S (≤0.5 μg/mL)	S (≤ 0.5 μg/mL)
Mupirocin	–	–	S (≤ 1 μg/mL)
Rifampicin	S (≤0.03 μg/mL)	S (≤0.03 μg/mL)	S (≤ 0,03 μg/mL)
Trimethoprim/Sulfamethoxazole	S (≤10 μg/mL)	S	R (≥ 320 μg/mL)
Teicoplanin	–	–	S (≤ 0.5 μg/mL)

MIC values and sensitivity to antibiotics were determined using Vitek®. The values which are above the resistance threshold for *Staphylococcus* spp. determined by EUCAST are marked in grey. R, resistant; I, susceptible, increased exposure; S, susceptible, standard dosing regimen.

**Table 4:** Antibiotic resistance of *M. luteus* strains.

Antibiotic	*M. luteus*	*M. luteus*	*M. luteus*	*M. luteus*
	School 1	School 2	School 3	ISS
Vancomycin	0.5 μg/mL	0.25 μg/mL	0.38 μg/mL	0.25 μg/mL
Ampicillin	<0.016 μg/mL	0.38 μg/mL	0.19 μg/mL	0.25 μg/mL
Piperacillin/Tazobactam	0.032 μg/mL	0.75 μg/mL	0.75 μg/mL	1.5 μg/mL
Cefotaxime	0.032 μg/mL	0.5 μg/mL	0.5 μg/mL	1 μg/mL
Meropenem	0.064 μg/mL	0.94 μg/mL	0.125 μg/mL	0.125 μg/mL
Linezolid	1 μg/mL	0.38 μg/mL	1.5 μg/mL	0.5 μg/mL
Penicillin G	<0.016 μg/mL	0.047 μg/mL	0.125 μg/mL	0.19 μg/mL

MIC values were determined using e-tests and microdilution assays. To determine MIC values indicating resistance PK/PD values “When there are no breakpoints” were used. The values which are above the resistance threshold for “When there are no breakpoints” determined by EUCAST are marked in grey.

*B. subtilis* school strains showed resistance to clindamycin, ciprofloxacin, and meropenem while *B. subtilis* ISS strains only showed resistance to ciprofloxacin (Table 2). *B. cereus* strains from the ISS all showed resistance to ciprofloxacin and meropenem, and *B. cereus* W2_B2a K2 additionally showed resistance to clindamycin.

*S. epidermidis* School showed resistance to tetracycline. *S. haemolyticus* ISS showed resistance to erythromycin. *S. aureus* School was an MRSA, hence being resistant to cefazolin and flucloxacillin, the first-choice antibiotics, as well as the other β-lactam antibiotics (Table 3).

*M. luteus* ISS showed resistance to cefotaxime and to piperacillin/tazobactam (Table 4). The determined MIC values were close to the respective breakpoints of 1 μg/mL for gram-positive bacteria against piperacillin/tazobactam and 0.5 μg/mL against cefotaxime (EUCAST, 2024).

*B. subtilis* school strains exhibited a higher number of antibiotic resistances than *B. subtilis* ISS strains. *S. haemolyticus* ISS and *S. epidermidis* School not did not show high antibiotic resistance. This is contraindicatory to our hypothesis, that ISS isolates may exhibit higher levels of antibiotic resistance. However, in comparison between all *M. luteus* isolates, only *M. luteus* exhibited slightly increased resistance to two antibiotics, which is in line with the above stated hypothesis. Hence, overall, no consistent trend was observed whether isolates recovered from Touch Arrays from the ISS exhibit higher antibiotic resistance than isolates recovered from school Touch Arrays. Additionally, since no isolate was resistant to all antibiotics, therapeutic options would remain available in the event of infection. Thus, despite the presence of antibiotic resistance, effective antibiotics would still be available for treatment in the event of infection.

### 3.2 Comparative characterization of isolates towards spaceflight-relevant conditions

To test whether space or school isolates might have an increased tolerance towards space-related factors such as X-ray irradiation, and desiccation we narrowed down the number of isolates. We selected *M. luteus* strains since we recovered three school isolates and one space isolate of *M. luteus* allowing us to compare school and space strains of one genus. Additionally, we selected three different *Staphylococcus* strains including the coagulase-negative *S. haemolyticus* ISS and *S. epidermidis* School. Moreover, we selected an environmental MRSA school strain, which was isolated in the scope of the citizen science projects of “Touching Surfaces” (Krämer et al., 2025). In order to assess whether resistance was increased of school and ISS strains, we tested type strains of the respective species of the German Strain Collection (DSMZ), namely *M. luteus* DSM 20030, *S. haemolyticus* DSM 20263, *S. epidermidis* DSM 20044, and *S. aureus* DSM 20231 alongside the space and school isolates.

### 3.3 Stress tolerance of *M. luteus* and *Staphylococcus* spp.

Desiccation tolerance was evaluated because built environments often present nutrient-limited, dry conditions. X-ray irradiation was examined since ionizing radiation levels are elevated on the ISS and in prospective space habitats compared to Earth. Hydrogen peroxide was selected for testing due to its use as a cleaning agent on the (ISS) and its relevance for assessing oxidative stress resistance.

#### 3.3.1 Desiccation

**Figure 1:**
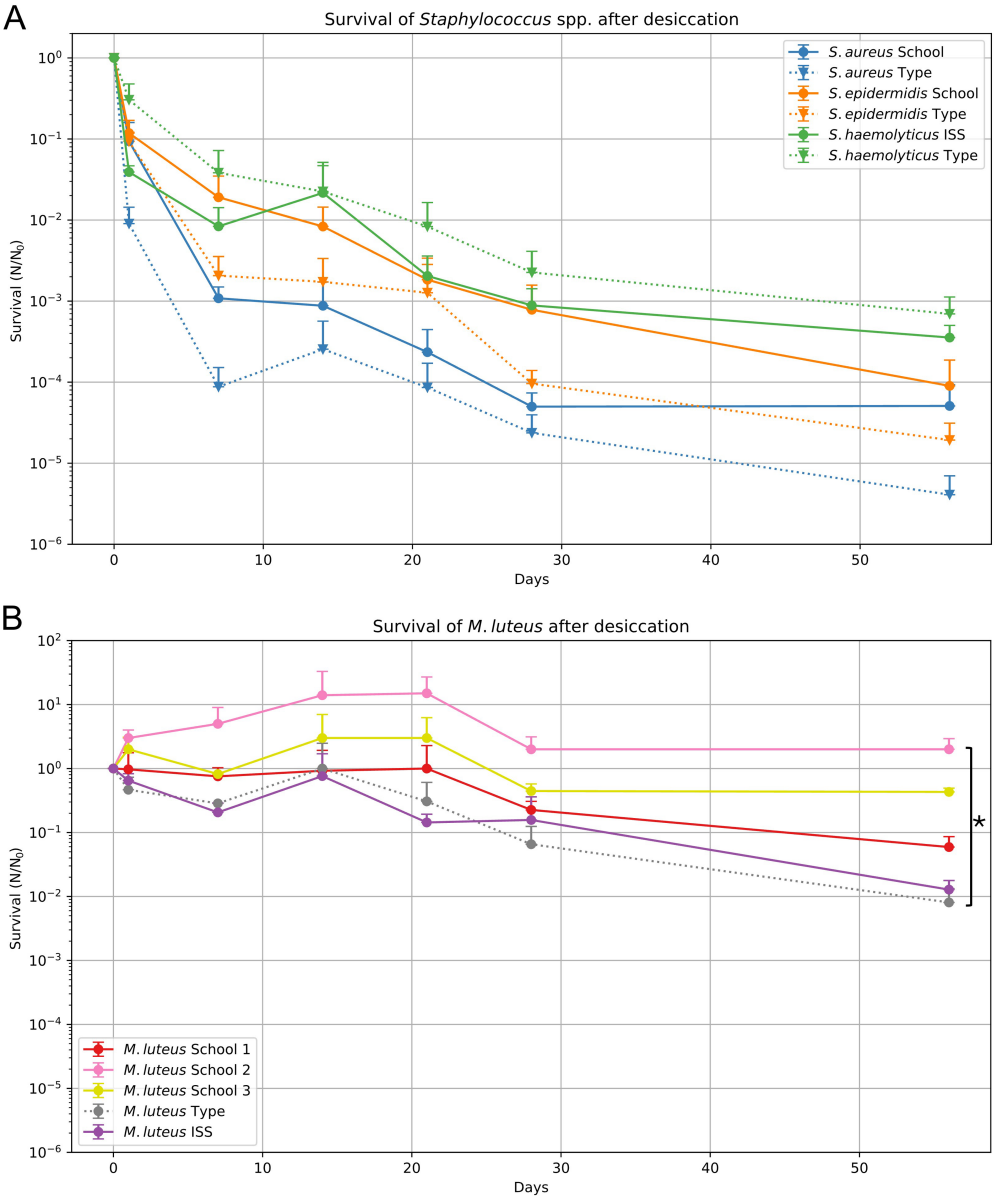
Desiccation tolerance of *Staphylococcus* spp. **(A)** and *M. luteus* **(B)** for 56 days. Survival fraction of *Staphylococcus* and *M. luteus* strains was determined by dividing the cell count after the respective treatment by the initial cell count (N/N_0_). The average of three independent experiments with three biological replicates each is given. Error bars represent the calculated standard deviation. Statistical significance was calculated between ISS and schools isolates and respective type strain of each species and is marked by an asterisk (*p < 0.05).

Given the potentially high desiccation tolerance of both school and space isolates, further investigation is essential to mitigate the risk of persistent bacterial strains in spacecraft. Strains were desiccated for up to 56 days. Survival of *Staphylococcus* strains and *M. luteus* strains after desiccation is given in Figure 1.

Survival of *S. aureus* School and *S. aureus* Type after desiccation was overall lower than for the other *Staphylococcus* species, and *S. epidermidis* strains showed the overall highest survival after desiccation. While survival of all *Staphylococcus* strains dropped with consecutive days of desiccation, growth was still detectable after desiccation for 56 days. Survival of space and school isolates did not differ significantly from survival of *Staphylococcus* type strains after 56 days (Figure 1A).

While survival of *M. luteus* ISS, *M. luteus* Type, and *M. luteus* School 1 declined over time, survival of *M. luteus* School 2, and *M. luteus* School 3 did not show a decline during desiccation, but actually cell count increased after desiccation. Desiccation tolerance of *M. luteus* ISS was not significantly higher than of *M. luteus* Type. However, survival after desiccation of *M. luteus* School 2 was significantly higher than survival of *M. luteus* Type (Figure 1B).

*S. aureus* School and *S. epidermidis* School demonstrated greater desiccation tolerance than their respective type strains, whereas *S. haemolyticus* ISS exhibited reduced desiccation tolerance compared to its type strain. All *M. luteus* strains displayed high desiccation tolerance, with *M. luteus* School 2 showing the highest level. These findings contradict the initial hypothesis that ISS strains would exhibit increased resistance.

#### 3.3.2 X-ray radiation tolerance

**Figure 2:**
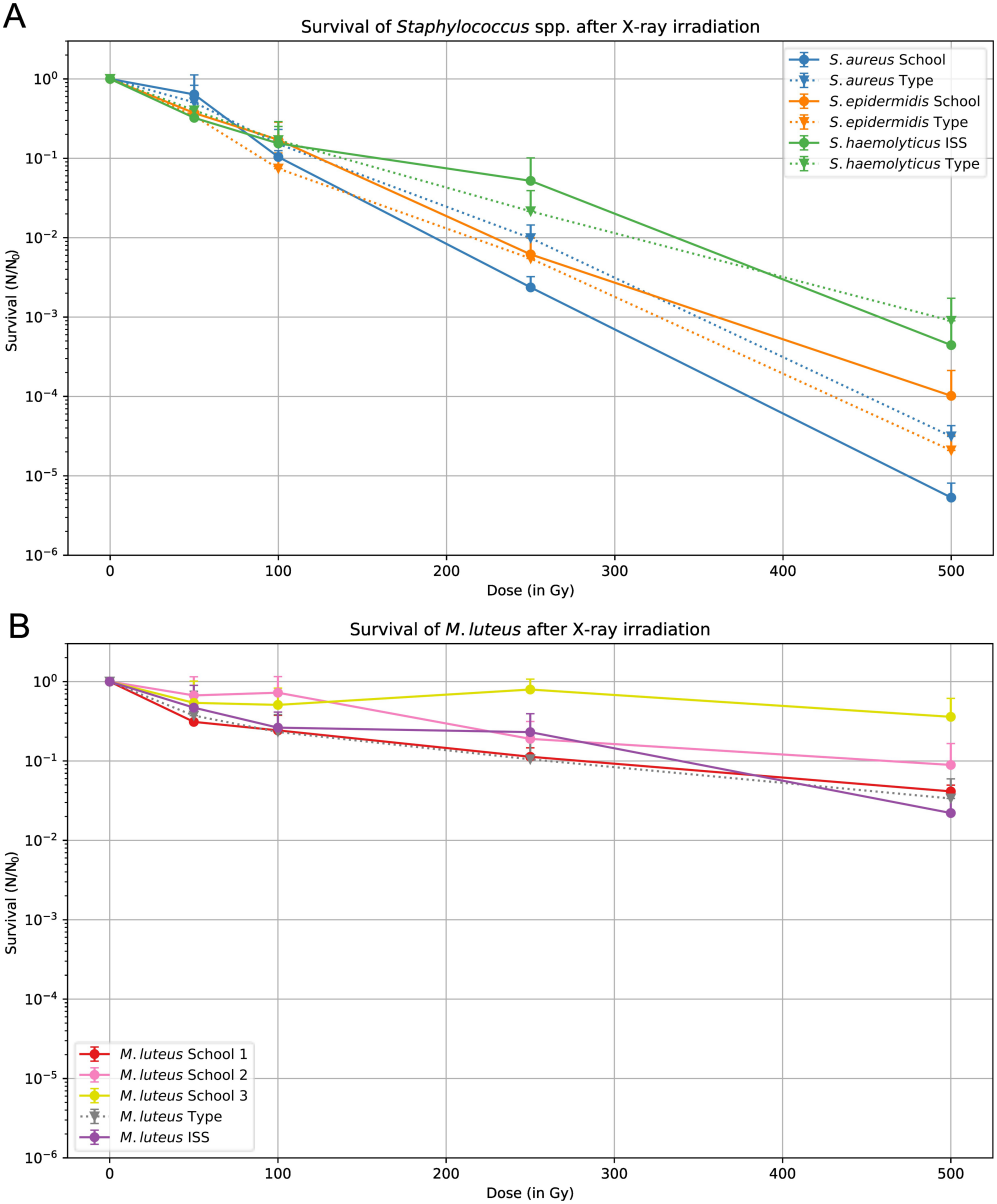
Survival of *Staphylococcus* spp. **(A)** and *M. luteus* **(B)** after X-ray irradiation. To determine survival of strains, cell counts after irradiation were divided by the initial cell count. Each datapoint represents the average of three independent experiments with three biological replicates each. Error bars show the calculated standard deviation. Statistical significance was calculated between the respective isolate and type strain of each species, but no significant differences were determined.

To determine whether ISS strains exhibited better survival to X-ray irradiation than type and school strains following prior exposure to increased radiation on the ISS, strains were exposed to X-ray irradiation at doses ranging from 50 Gy to 500 Gy. Survival of *Staphylococcus* and *M. luteus* strains is displayed in Figure 2.

Survival of *S. haemolyticus* ISS (LD_90_ = 121.7 Gy *σ* 54.5 Gy) and *S. haemolyticus* Type (LD_90_ = 134.8 Gy σ 36.7 Gy) was highest of all *Staphylococcus* strains, but did not differ significantly between ISS and type strain. The survival of *S. epidermidis* School (LD_90_ = 115.4 Gy σ 19.8 Gy) after exposure to 500 Gy was higher than survival of the respective type strain (LD_90_ = 99.2 Gy σ 14.3 Gy), but we did not find statistical significance. Survival of *S. aureus* School (LD_90_ = 100.4 Gy σ 12.6 Gy) strain after exposure to 500 Gy was lower than survival of *S. aureus* Type (LD_90_ = 113.8 Gy σ 23.7 Gy), but did not differ significantly (Figure 2A). The LD_90_ of *Staphylococcus* spp. was not statistically different between type strains and isolates.

Survival of *M. luteus* strains after exposure to X-ray irradiation was overall high with a 1-log to 2-log reduction, with *M. luteus* School 3 exhibiting the highest survival after X-ray irradiation. However, we did not find statistically significant differences in survival after X-ray irradiation between *M. luteus* Type and school/ISS strains (Figure 2B). Overall survival after X-ray irradiation of *M. luteus* isolates was higher than of *Staphylococcus* isolates. Due to the high survival rate of *M. luteus* isolates, LD_90_ could not be calculated accurately.

X-ray irradiation tolerance varied among the strains. *S. haemolyticus* ISS and *S. aureus* School were less resistant to radiation than their respective type strains, whereas *S. epidermidis* School demonstrated greater resistance than its type strain. *M. luteus* School 3 exhibited the highest X-ray tolerance. These results do not support the hypothesis that ISS strains have adapted to increased ionizing radiation exposure.

#### 3.3.3 Hydrogen peroxide

**Figure 3:**
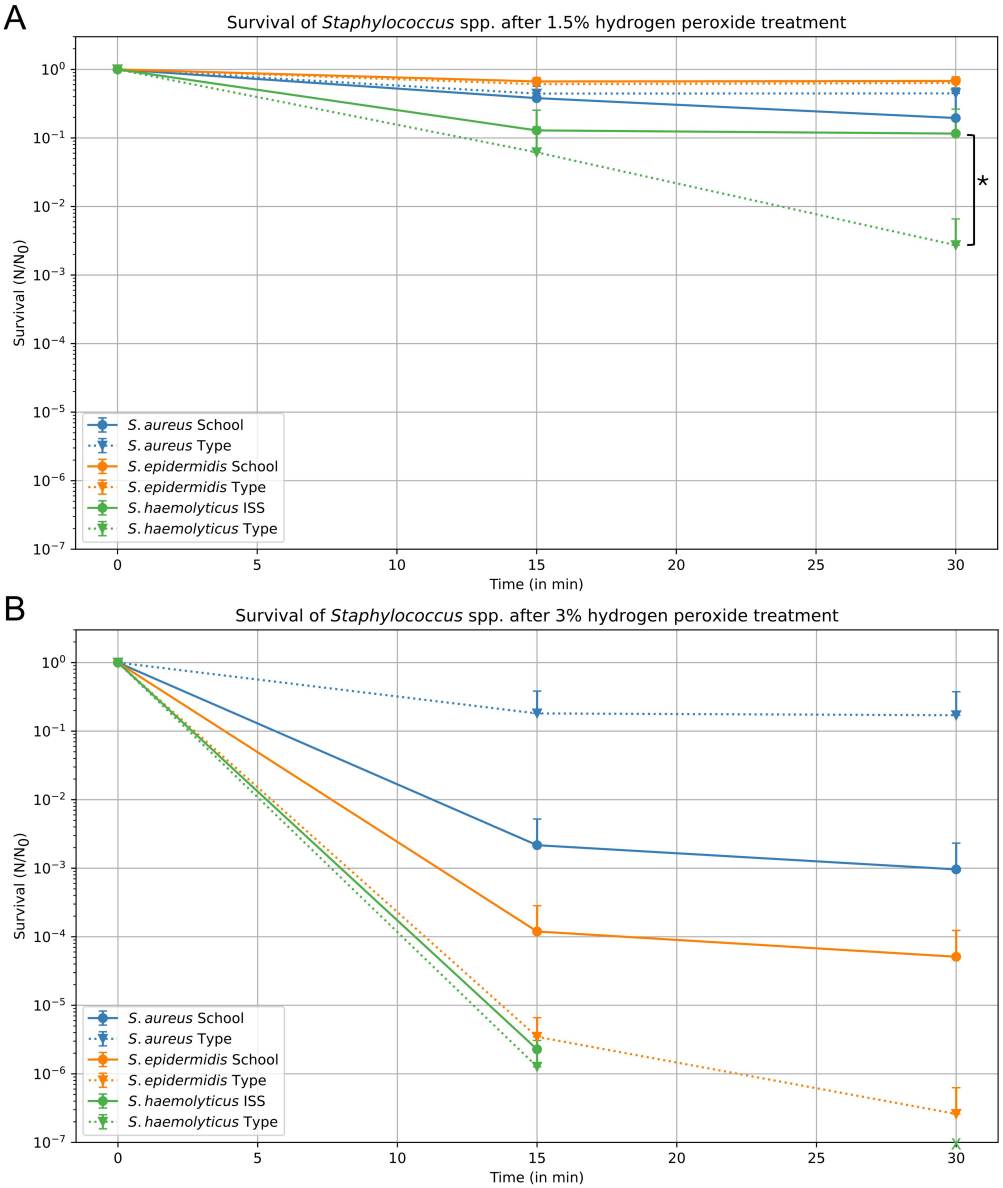
Survival of *Staphylococcus* strains after exposure to **(A)** 1.5% and **(B)** 3% hydrogen peroxide. Survival fraction was determined by dividing the respective cell count after treatment by the initial cell count. The initial cell count was set to 10^8^ CFU/mL and datapoints represent the average of three independent experiments with three biological replicates each. Error bars represent the standard deviation. Statistical significance was calculated between the respective isolate and type strain of each species and is marked by an asterisk (*p < 0.05). No detected survival is indicated by an X.

**Figure 4:**
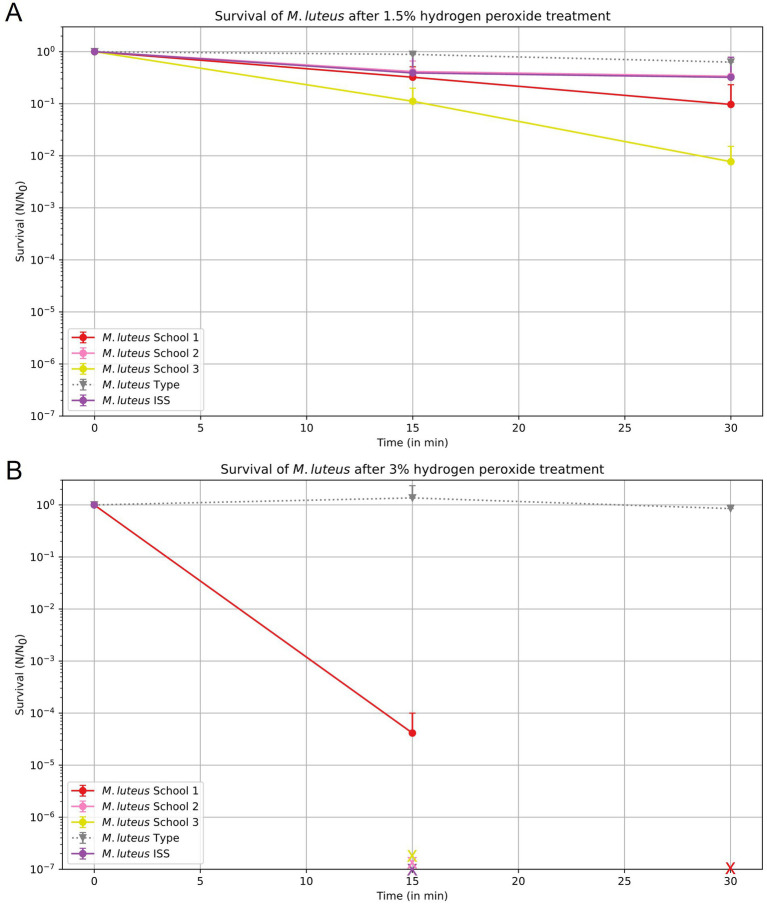
Survival of *M. luteus* strains after exposure to **(A)** 1.5% and **(B)** 3% hydrogen peroxide. Survival fraction was determined by dividing the respective cell count after treatment by the initial cell count. The initial cell count was set to 10^8^ CFU/mL and datapoints represent the average of three independent experiments with three biological replicates each. Error bars show the calculated standard deviation. No detected survival is indicated by an X. Statistical significance was calculated between the respective isolate and type strain of each species, but no statistically significant difference was determined.

Cleaning onboard the ISS necessitates specialized methods and equipment, as the use of flammable agents or free-flowing liquids poses significant safety and operational challenges in a microgravity environment. As in every built habitat, the ISS crew has to regularly clean their home and environment, which on the ISS, is performed using hydrogen peroxide wipes (Viroxtechnologies, 2018). To test whether space or school isolates showed increased resistance to hydrogen peroxide treatment, isolates were exposed to 1.5% hydrogen peroxide and 3% hydrogen peroxide (Figures 3, 4). A concentration of 3% hydrogen peroxide was included as it is a commercially commonly available concentration. The 1.5% concentration was included to assess differences in reaction rates. These concentrations also align with standard laboratory testing conditions and allow comparison with a previous study on genotypic and phenotypic differences in *Staphylococcus capitis* isolates (Siems et al., 2022).

*S. epidermidis* strains showed no reduction in survival after 30 min treatment with 1.5% hydrogen peroxide, but survival of *S. epidermidis* School was significantly higher after treatment with 3% hydrogen peroxide after 30 min (*p* = 0.00875). Survival of *S. epidermidis* Type was below the detection level after 30 min exposure to 3% hydrogen peroxide.

Strains of *S. aureus* showed less than 1-log reduction after 30 min exposure to 1.5% hydrogen peroxide, but survival of *S. aureus* School was higher than survival of *S. aureus* Type after 15 min and 30 min exposure to 3% hydrogen peroxide.

After treatment with 1.5% hydrogen peroxide survival of *M. luteus* School 1 decreased by 1-log and survival of *M. luteus* School 3 decreased by 2-log compared to *M. luteus* Type. However, *M. luteus* Type, *M. luteus* ISS and *M. luteus* School 2 all survived treatment with 1.5% hydrogen peroxide for up to 30 min with less than 1-log decrease in survival.

*M. luteus* ISS, *M. luteus* School 2, and *M. luteus* School 3 did not survive treatment of 3% hydrogen peroxide for 15 min or 30 min. *M. luteus* School 1 survived 15 min treatment with 3% hydrogen peroxide with a 4-log reduction. *M. luteus* Type displayed survival without reduction after 15 min and 30 min hydrogen peroxide treatment.

*S. epidermidis* School exhibited a slight increase in hydrogen peroxide tolerance. *S. aureus* Type demonstrated greater hydrogen peroxide tolerance than *S. aureus* School. *S. haemolyticus* ISS showed a significant increase in hydrogen peroxide tolerance. Among *M. luteus* strains, *M. luteus* Type was the most resistant. The results are inconsistent, as the ISS isolate of *S. haemolyticus* showed increased tolerance, but this was not observed in *M. luteus*. Overall, there is no clear evidence supporting the hypothesis that ISS strains are more adapted to oxidative stress.

### 3.4 Indicators of surface attachment

#### 3.4.1 Morphology

**Figure 5:**
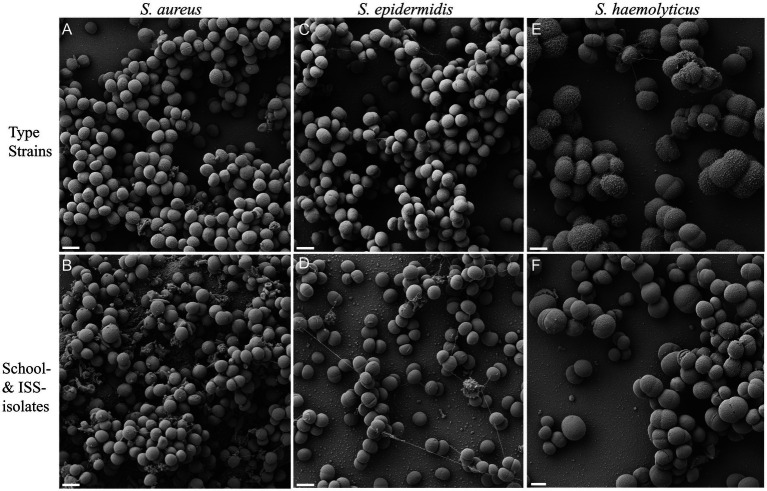
SEM images of *Staphylococcus* spp. after 24 h of static incubation. The top images represent type strains of **(A)** *S. aureus*, **(C)** *S. epidermidis*, **(E)** *S. haemolyticus*. Bottom images represent school and ISS isolates of the respective species, **(B)** *S. aureus* School, **(D)** *S. epidermidis* School, **(F)** *S. haemolyticus* ISS. The scale bar corresponds to 1 μm.

**Figure 6:**
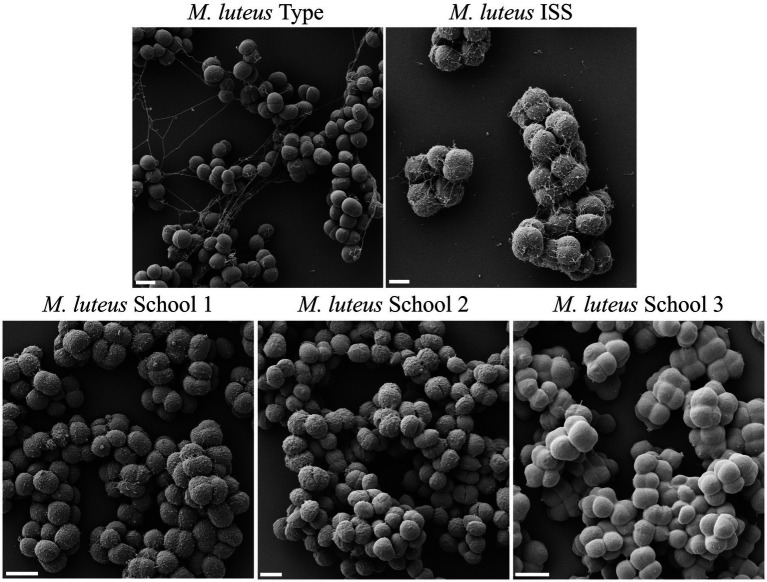
SEM images of *M. luteus* isolates after 24 h static incubation on glass discs. The scale bar is equivalent to 1 μm.

An overview of the phenotypic appearance of the tested *Staphylococcus* and *M. luteus* isolates is given in Supplementary Figure 1. For a more in-depth analysis, we acquired SEM images of *Staphylococcus* (Figure 5) and *M. luteus* (Figure 6) isolates.

Overall, *S. aureus* Type and *S. aureus* School showed comparable morphology, particularly in regard to their surface attachment and structure (Figures 5A,B). Filamentous structures were observed for *S. epidermidis* Type and *S. epidermidis* School, which differed from the morphologies of the other *Staphylococcus* spp. (Figures 5C,D). *S. haemolyticus* Type and *S. haemolyticus* ISS also showed structural similarities, including a pronounced size and shape variability, which was evident both between the two strains and within the respective sample (Figures 5E,F).

The different isolates of *M. luteus* depicted in Figure 6 show different morphologies. *M. luteus* Type formed dense aggregates, and filamentous structures were visible, indicating the presence of early-stage developing biofilm matrix. *M. luteus* School 1 exhibited tightly attached cellular structures with close cell–cell associations, which were found bundled together on the surface. *M. luteus* School 1 and *M. luteus* School 2 adhered strongly to the surface and showed the highest degree of surface colonization, with the largest number of cells observed. *M. luteus* ISS occurred predominantly in tetrad arrangements, which were embedded within a putative early-stage forming biofilm matrix.

#### 3.4.2 Biofilm formation determined by crystal violet assay

**Figure 7:**
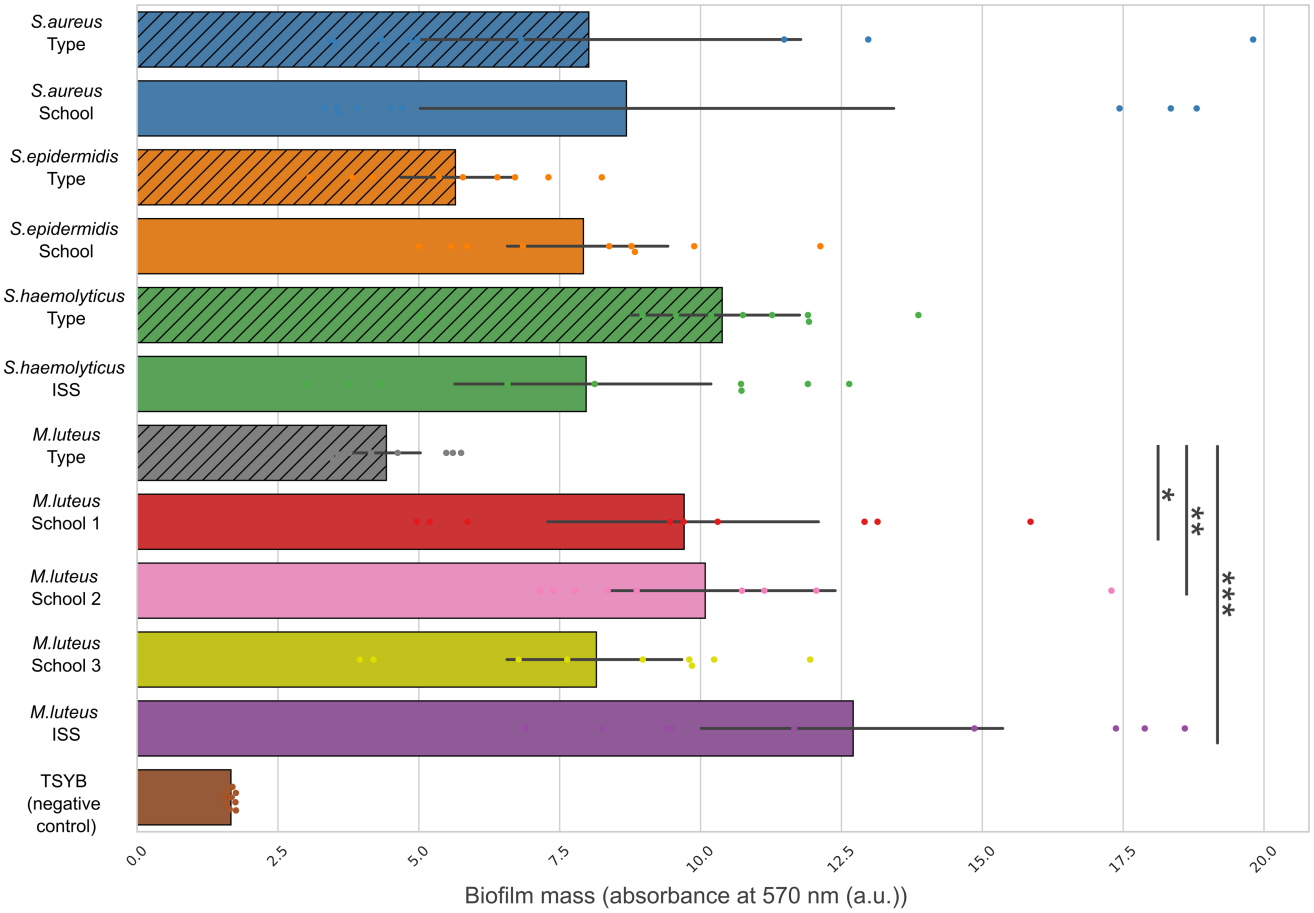
Biofilm formation of *Staphylococcus* spp. and *M. luteus* strains. To investigate early biofilm formation of *Staphylococcus* and *M. luteus* strains, the semiquantitative crystal violet assay was performed. The strains were statically incubated for 24 h at 37 °C before staining with crystal violet. Biofilm formation is proportional to absorption at 570 nm and was determined in three independent experiments with three biological replicates each with three technical replicates each. TSYB medium was used as a negative control for both *Staphylococcus* spp. and *M. luteus*. Statistical significance was tested between the school/space isolates and the respective type strain and is indicated by an asterisk (*p < 0.05, **p < 0.01, ***p < 0.001).

Early-stage biofilm formation was determined by the semi-quantitative crystal violet assay and is shown in Figure 7 for *Staphylococcus* and *M. luteus* strains.

While early-stage biofilm formation of *S. aureus* School was higher than of the respective type strain, this difference was not statistically significant. However, biofilm formation of *S. epidermidis* School was significantly higher (*p* = 0.0319) compared to biofilm formation of *S. epidermidis* Type. Biofilm formation of *S. haemolyticus* Type was higher compared to *S. haemolyticus* ISS, but again we did not find a statistically significant difference (Figure 7).

Early-stage biofilm formation of all *M. luteus* school and ISS strains was higher than biofilm formation of *M. luteus* Type. Biofilm formation of *M. luteus* School 2 (*p* = 0.008) and *M. luteus* School 1 (*p* = 0.013) was significantly higher than biofilm formation of *M. luteus* Type. ISS strain *M. luteus* ISS (*p* < 0.001) showed the highest biofilm formation of all isolates (Figure 7). Among *M. luteus* strains, *M. luteus* School 3 demonstrated the smallest increase in biofilm formation relative to *M. luteus* Type, although its biofilm production was nearly double that of *M. luteus* Type. Biofilm formation by *M. luteus* School 1 and *M. luteus* School 2 was on a similar level, but both were slightly higher than that of *M. luteus* School 3. Overall, biofilm formation by *M. luteus* ISS was the highest, showing nearly a three-fold increase in biofilm formation compared to *M. luteus* Type.

All isolates of *Staphylococcus* spp. and *M. luteus* demonstrated higher biofilm formation compared to their respective type strains. *M. luteus* ISS exhibited the greatest capacity for early-stage biofilm formation. These findings are consistent with the hypothesis that surface attachment and biofilm formation are enhanced in ISS isolates, although similar trends were observed in other surface-derived isolates.

A comparative overview of survival after stress exposure and early-stage biofilm formation is given in Table 5.

**Table 5:** Comparative overview of survival after stress exposure and early-stage biofilm formation.

Screening	*S. aureus*		*S. epidermidis*		*S. haemolyticus*		*M. luteus*				
	School	Type	School	Type	ISS	Type	ISS	School 1	School 2	School 3	Type
28 days desiccation	++	++	+++	++	+++	+++	++++	++++	++++	++++	+++
56 days desiccation	++	+	++	++	++	++	+++	+++	+++	++++	+++
X-ray irradiation	+	++	++	++	++	++	+++	+++	+++	++++	+++
30 min with 1.5% hydrogen peroxide	++++	++++	++++	++++	++++*	+++	++++	++++	++++	+++	++++
30 min at 3% hydrogen peroxide	++	++++	++	+	−	−	++++	−	−	−	++++
Early-stage biofilm formation	++	++	++	+	++	++	++++***	+++*	+++**	++	+

For stress exposure, survival is indicated through reduction in log (− = no survival detected, + = <5-log to 7-log, ++ = <3-log to 5-log, +++ = <1-log-3log, ++++ = = < 1-log) and for biofilm formation corresponding values of absorbance at 570 nm (in a.u.) are given (− = <2.5, + = 5, ++ = 7.5, +++ = 10, ++++ = 12.5). Green marked fields indicate highest survival rate or biofilm formation of the respective strain compared between each species. Detected significance is indicated by asterisks (*p < 0.05, **p < 0.01, ***p < 0.001).

### 3.5 Genome analysis

**Figure 8:**
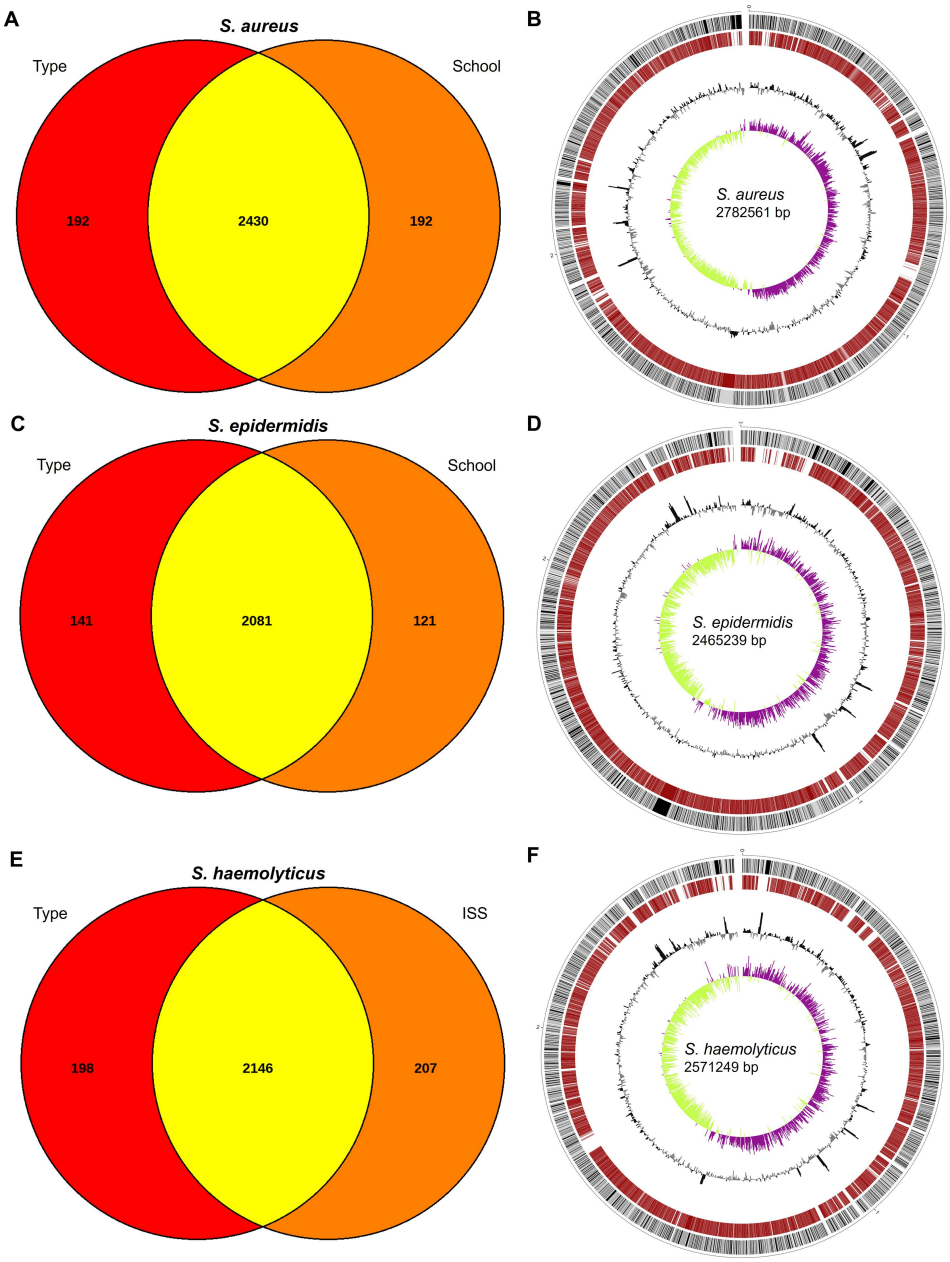
Comparative genome analysis of *Staphylococcus* strains. **(A,C,E)** Venn diagrams of shared genes (yellow) and singletons of the respective *Staphylococcus* spp. Singletons of the respective type strains are depicted in red, and singletons of the respective isolates are depicted in orange. **(B,D,F)** Comparative circular genome plots of the respective type strain and isolate of each *Staphylococcus* species. From outward to inward are shown: Coding genes in black, core genome in red, GC content (above mean in black, below mean in dark grey), and GC skew (above mean in purple, below mean in green). Figures were created using Edgar 3.0 (Dieckmann et al., 2021).

To characterize the genomic variation across the isolates, whole genomes of all strains were sequenced using Oxford Nanopore. Venn diagrams were used to visualize shared genes and singletons of the different *Staphylococcus* spp., and visualization of the circular genomes in respect to the type strains was performed using Edgar 3.0 (Dieckmann et al., 2021) and are shown in Figure 8. *S. aureus* Type and *S. aureus* School shared 2,430 coding DNA sequences (cds), and each had 192 singletons (Figure 8A), and showed overall high homology with gaps within the core genome of 13 kb and a larger gap of 42 kb with only some homology in between (Figure 8B). *S. epidermidis* Type and *S. epidermidis* School shared the majority of cds (2,081), and *S. epidermidis* Type had 141 singletons, while *S. epidermidis* School had 121 singletons (Figure 8C). Plotting of the circular genome showed overall high genomic homology with gaps from < 10 kb to 18 kb in the core genome of *S. epidermidis* Type and *S. epidermidis* School (Figure 8D). *S. haemolyticus* Type displayed 198 singletons, while *S. haemolyticus* ISS showed 207 singletons, both shared 2,146 cds. (Figure 8E). Overall homology of the core genome *S. haemolyticus* ISS and *S. haemolyticus* Type showed several gaps <10 kb, 5 gaps of 10–20 kb, and 2 gaps of <20 kb (~25 kb, ~34 kb) (Figure 8F).

Investigation of antimicrobial resistance and virulence genes was performed using ABRicate, and genes with a query coverage and identity above 80% are listed in Supplementary Table 3 for *Staphylococcus* spp. and in Supplementary Table 4 for *M. luteus.* An overview of detected AMR and virulence genes according to the different databases is given in Table 6 and Supplementary Tables 5–8. The highest number of AMR and virulence genes was retrieved for *S. aureus* strains, with a higher number of AMR and virulence genes for *S. aureus* School than for *S. aureus* Type. Among the genes of *S. aureus* School not shared with *S. aureus* Type was *mecA* (Table 6; Supplementary Tables 3, 5). In the genome of *S. epidermidis* Type genes involved in β-lactam resistance (e.g., *blaI, blaZ*) were detected, but not in the genome of *S. epidermidis* School (Table 6; Supplementary Tables 3, 6). Genome analysis of *S. haemolyticus* ISS revealed the presence of genes involved in macrolide resistance (e.g., *mphC, msrA*), which were not present in the genome of *S. haemolyticus* Type (Table 6; Supplementary Tables 3, 7).

**Table 6:** Overview of detected AMR and virulence genes across different used databases.

Strain	Total number of detected genes	Detected AMR and virulence genes
*S. aureus* Type	78	aac(3), adsA, aph(3′)-I, arlR, arl, cap8A, cap8B, cap8C, cap8D, cap8E, cap8F, cap8G, cap8L, cap8M, cap8N, cap8O, cap8P, clfA, clfB, coa*, dhaP, ebp, esaABC, essABC, esxAB, fnbA, fnbB*, fosB*, geh, hlb, hld, hlgABC, hly/hla, hysA, icaABCDR, isdABCDEFG, lip, lmrS, lukF-PV, map, mepABR, mgrA, norAB, rlmH, sbi, sdrCDE, spa, srtB, sspABC, tet(38), vWbp
*S. aureus* School	98	aac(3), aac(6′)-I*, adsA, aph(2″)-Ia2*, aph(2″)-Ih*, aph(3′)-I, arlR, arlS, aur, blaI*, blaR*, blaZ*, cap8A, cap8B, cap8C, cap8D, cap8E, cap8F, cap8G, cap8H, cap8I, cap8J, cap8K, cap8L, cap8M, cap8N, cap8O, cap8P, chp*, clfA, clfB, dhaP, ebp, erm(B)*, esaABC, essABC, esxAB, fnbA, geh, hlb, hld, hlgABC, hly/hla, hysA, icaABCDR, isdABCDEFG, lip, lmrS, lukF-PV, map, mecA*, mepABR, mgrA, norAB, rlmH, sak*, sbi, scn*, sdrCDE, sec*, sell*, spa, srtB, sspABC, tet(38), tsst-1*, vWbp
*S. epidermidis* Type	14	aph(3′)-I, blaI*, blaR*, blaZ*, dfrC, fosB, mgrA, norA
*S. epidermidis* School	8	aph(3′)-I, dfrC, fosB, mgrA, norA, rlmH*
*S. haemolyticus* Type	3	*aph(3′)-I, mgrA, rlmH*
*S. haemolyticus* ISS	11	aph(3′)-I, mgrA, mphC*, msrA*, qacAB*, qacR*, rlmH
*M. luteus* Type	6	blaTEM*, aph(3′)
*M. luteus* School 1	3	*aph(3′)*
*M. luteus* School 2	6	blaTEM*, aph(3′)
*M. luteus* School 3	6	blaTEM*, aph(3′)
*M. luteus* ISS	3	*aph(3′)*

Virulence and AMR related genes were detected via ABRicate using megares, VFDB, resfinder and NCBI. The total number of genes detected across the different databases is given, but can include multiple detection of one gene. Detected AMR and virulence genes are given for each strain, and genes which were not shared between all strains of each respective species, but were exclusive to the respective strain, are indicated by an asterisk (*).

**Figure 9:**
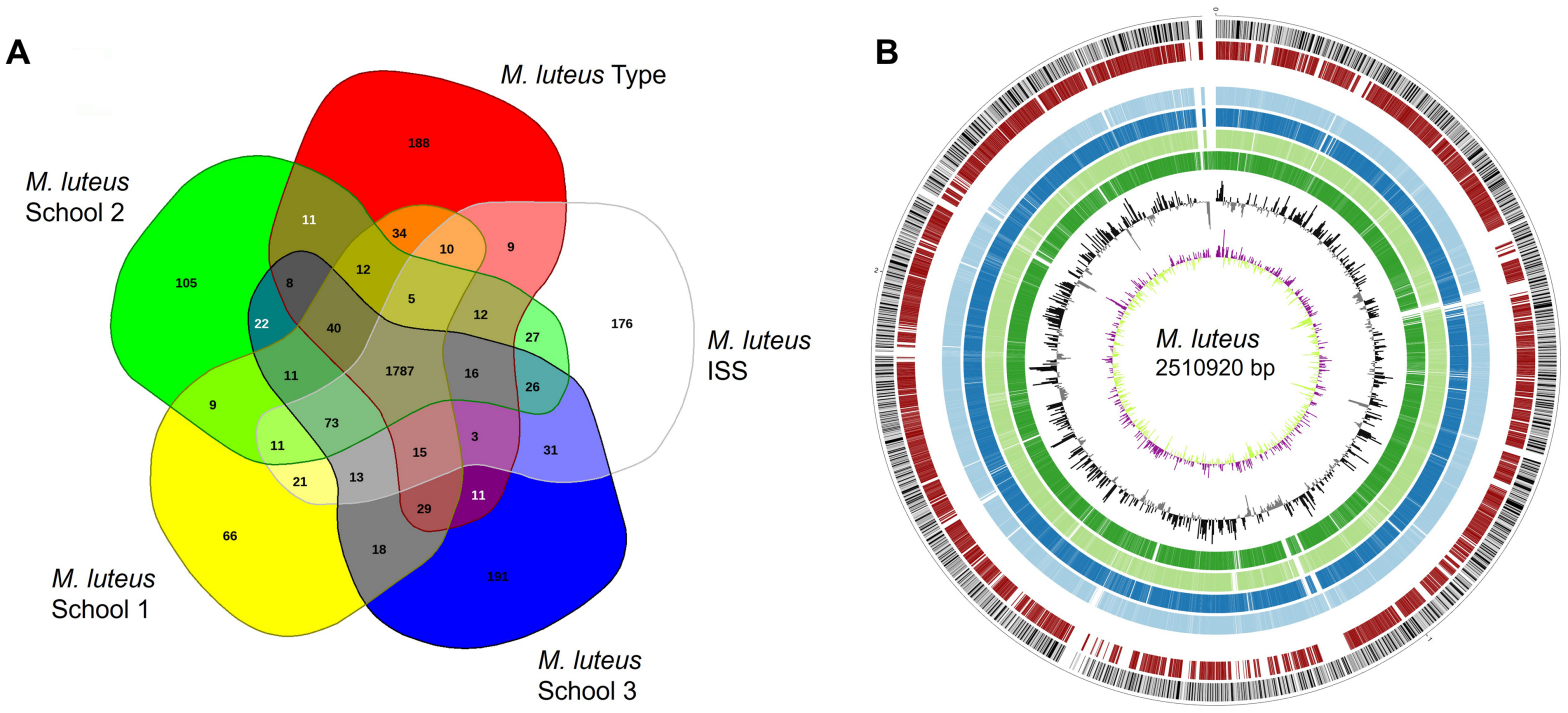
Comparative genome analysis of *M. luteus* strains. **(A)** Venn diagram of shared genes and singletons of *M. luteus* strains. *M. luteus* Type (red), *M. luteus* School 1 (yellow), *M. luteus* School 2 (green), *M. luteus* School 3 (blue), *M. luteus* ISS (grey). **(B)** Comparative circular genome plot of *M. luteus* strains. *M. luteus* Type was set as reference. Legend from outward to inward: coding DNA sequences of *M. luteus* in black, core genome in red, pairwise alignment of *M. luteus* School 1 (light blue), *M. luteus* School 2 (dark blue), *M. luteus* School 3 (light green), and *M. luteus* ISS (dark green) with *M. luteus* Type; GC content (above mean in black, below mean in dark grey), and GC skew (above mean in purple, below mean in green). Figures were created using Edgar 3.0 (Dieckmann et al., 2021).

*M. luteus* isolates were defined as *Micrococcus yunnanensis* after whole genome sequencing. However, *M. yunnanensis* is a heterotypic synonym for *M. luteus* and was reclassified to *M. luteus* since the average identity of nucleotides and amino acids were found to be greater than the threshold for species delineation (Huang et al., 2019). The use of *M. yunnanensis* is not recommended for medical use according to the Leibniz Institute- German Collection of Microorganisms and Cell Cultures (DSMZ, 2026). Hence, the isolates were referred to as *M. luteus.* Comparative analysis of the shared and unique cds of *M. luteus* strains revealed that all *M. luteus* strains shared 1,787 cds, *M. luteus* School 1 had the lowest number of singletons (66) and *M. luteus* School 3 had the highest number of singletons (191). The number of shared cds was not higher among school isolates compared to overall shared cds (Figure 9A). Comparison of the circular genome plots of *M. luteus* strains showed overall great homology. While there were some gaps in the core genome, the comparative circular genome plot revealed two regions of divergence. One region showed differently sized gaps between the isolates and Type strain ranging from ~8 kb (*M. luteus* School 3) to ~16 kb (*M. luteus* School 1). Additionally, in one region the genome from *M. luteus* ISS showed a gap of ~22 kb compared to the other *M. luteus* strains (Figure 9B).

Investigation of AMR and virulence genes in *M. luteus* strains showed only two genes, namely *blaTEM*, which encodes for β-lactam resistance and was present in all *M. luteus* strains and *aph(3′)*, which encodes for a kanamycin kinase and was present in *M. luteus* Type, *M. luteus* School 2, and *M. luteus* School 3 (Table 6; Supplementary Tables 4, 8).

Comparative assessment of genotypic features showed more genes related to AMR and virulence in *S. aureus* School and *S. haemolyticus* ISS compared to their type strains. *S. epidermidis* had fewer AMR and virulence genes than its type strain. *M. luteus* strains had a low number of virulence and AMR genes. *M. luteus* Type, School 2, and School 3 carried *blaTEM*, unlike *M. luteus* ISS and School 1. Hence, different indications about the hypothesis that ISS strains harbor more AMR and virulence genes were found here.

Overall, high genomic similarity among isolates with limited strain-specific variation in gene content does not indicate habitat- specific association and adaptation on a genomic level.

## 4 Discussion

Microbial resistance is a critical concern in many habitats, since selective pressures and microbial adaptation allow resistant strains to persist and spread. We, therefore, investigated the antibiotic resistance and survival to space-relevant stressors such as desiccation, X-ray irradiation, and hydrogen peroxide treatment of strains isolated from surfaces exposed to frequent touching in schools and the ISS, to determine whether the habitat background influences their potential for antibiotic resistance. Additionally, we compared early-stage biofilm formation of school and ISS isolates and investigated possible resistance genes through genome characterization.

During missions, astronauts can develop infections caused by opportunistic pathogens, requiring careful microbiological diagnosis (Boschert et al., 2025). *Bacillus* spp. have been regularly isolated from the ISS and also from medical equipment for blood sampling (Yenikeyev et al., 2022; Venkateswaran et al., 2017). *B. subtilis* isolates from schools showed resistance to three out of the five tested antibiotics, namely clindamycin, ciprofloxacin, and meropenem. Both *B. subtilis* isolates from the ISS were resistant to ciprofloxacin. *B. subtilis* is a relevant organism for space microbiology, due to the resistance of its spores against multiple space-related conditions (Wassmann et al., 2012; Cortesão et al., 2019). Transcriptome analysis of *B. subtilis* from two ISS missions also revealed differential gene expression with the upregulation of genes related to biofilm formation, biotin and arginine biosynthesis and toxin production in spaceflight samples (Morrison et al., 2019). Additionally, we tested antibiotic resistance of three *B. cereus* isolates from the ISS of which all were resistant to ciprofloxacin and meropenem. One *B. cereus* isolate was additionally resistant to clindamycin. *B. cereus* is an opportunistic pathogen and the causative agent of many gastrointestinal infections. Its S-layer, a special surface structure, plays an important role in the adhesion to host cells which contributes to its pathogenicity and has been shown to increase resistance to gamma radiation (Kotiranta et al., 1999; Kotiranta et al., 2000; Gerbino et al., 2015). Non-toxin-producing *B. cereus* strains closely related to *B. anthracis* have been identified on the ISS and have been shown to accumulate genetic variations unique to the ISS environment (Quagliariello et al., 2022; Venkateswaran et al., 2017). However, none of the *Bacillus* isolates were resistant to vancomycin and linezolid, leaving viable treatment options available. Another study found that the virulence of *Bacillus* isolates was low in confined habitats, but these strains may be involved in the spread of virulence genes via horizontal gene transfer (Timmery et al., 2011). Other studies which investigated antimicrobial susceptibility of *Bacillus* spp. isolated from milk, food or retail markets found diverse resistance profiles across the *Bacillus* genus (Hu et al., 2021; Adamski et al., 2023; Fiedler et al., 2019) highlighting the need for antimicrobial surveillance in food industries, but also in human habitats such as space habitats which are confined and often have multiple-purpose areas.

However, it is particularly important to distinguish between infection and intoxication by *Bacillus*- associated toxins: In foodborne diseases, the toxin is the main trigger of the symptoms, while the pathogen itself is of less therapeutic relevance (Jovanovic et al., 2021). Hence, antibiotic treatment is often not effective since antibiotics do not neutralize toxins.

In our study, we tested staphylococci because they thrive in a variety of environments, including clinically and space-relevant habitats. Among other things, their metabolic adaptability enables them to survive in many different habitats, giving them the versatility and persistence to have a global health impact and demonstrated resilience (Onyango and Alreshidi, 2018). Moreover, as they are part of the human physiological skin flora, they will inevitably accompany human space exploration.

We decided to include *S. haemolyticus* due to its nature of being a skin commensal, but also an emergent danger in clinical environments (Czekaj et al., 2015). Although *S. haemolyticus* is coagulase-negative and therefore lacks virulence factors compared to *S. aureus*, it is among the most commonly isolated coagulase-negative *Staphylococcus* in clinical cases (Teeraputon et al., 2017; Nanoukon et al., 2017; Takeuchi et al., 2005). Studies have shown that *S. haemolyticus* can provide a pool of antibiotic resistance genes to other staphylococci such as *S. aureus* thereby contributing to increasing AMR (Fluit et al., 2013). Antibiotic susceptibility testing of the *S. haemolyticus* ISS strain revealed resistance to erythromycin, a protein synthesis-inhibiting antibiotic active against a variety of gram-positive bacteria. In the genome of *S. haemolyticus* ISS genes encoding for resistance to macrolide antibiotics, including erythromycin, were also detected. Within the *Staphylococcus* genera, erythromycin and erythromycin-inducible resistance have been commonly reported since the 1950s (Hobson, 1954; Mahfouz et al., 2023). A previous study found, that strains of *S. haemolyticus* from the ISS did not show an increased resistance to antibiotics (Schiwon et al., 2013), which is in line with our findings indicating that there are still treatment options left due to the susceptibility to other antibiotics. One study revealed that 75% of isolated *S. haemolyticus* from patients of a Brazilian hospital showed multidrug-resistance (Barros et al., 2012). High multi-drug resistance in *S. haemolyticus* can additionally contribute to the emergence of more multi-resistant *S. aureus* strains, for example through horizontal gene transfer (Cavanagh et al., 2014; Fluit et al., 2013). Another study used mating experiments to demonstrate that a *S. haemolyticus* ISS isolate was indeed able to transfer resistance genes to *Enterococcus faecalis* and *S. aureus* (Schiwon et al., 2013). In our study, *S. haemolyticus* ISS did not show an increased resistance to X-ray irradiation, desiccation, or hydrogen peroxide treatment compared to *S. haemolyticus* Type. To date, we have not been able to identify other studies which have tested resistance of *S. haemolyticus* to the previously named stressors.

Among the different *Staphylococcus* species *S. aureus* is the most virulent. Hence, we decided to test resistance of an MRSA isolate from the citizen science part of the Touching Surfaces ISS experiment. Analysis of whole genomes showed that *S. aureus* strains harbored the largest amount of AMR and virulence genes compared to the other tested species. MRSA is one of the most common clinical isolates and a leading cause of bacterial infections (Appelbaum, 2006). While *S. aureus* colonizes the nasal mucosa of approximately 20–40% of the population in Germany (Becker et al., 2017), *S. aureus* can also cause severe infections (Chambers and DeLeo, 2009). In contrast to the genome of *S. aureus* Type, the genome of *S. aureus* School harbored *mecA*. Due to the acquisition of the staphylococcal cassette chromosome *mec* (SSC*mec*) with genes encoding for proteins aiding in antibiotic resistance, MRSA is resistant to most β-lactam antibiotics and enables the acquisition of additional resistance genes rendering MRSA a threat in the current antimicrobial resistance crisis (Ito and Hiramatsu, 1998; Noto et al., 2008). Detection of the *mecA* gene in *S. aureus* School indicates broad resistance to β-lactam antibiotics, consistent with antibiotic resistance testing of *S. aureus* School, which demonstrated susceptibility to only 39% of the tested antibiotics. This limited susceptibility leaves few available treatment options in the event of infection and precludes the use of conventional first-line therapies, such as cefazolin or flucloxacillin. Besides its spread as a nosocomial pathogen, MRSA has also become a problem in community-acquired infections (Kluytmans-VandenBergh and Kluytmans, 2006). The absence of *pvl*, which encodes Panton-Valentine leucocidin (PVL), in *S. aureus* strains is a favorable finding, as PVL-positive strains are associated with recurrent skin and soft tissue infections that are difficult to treat (David Michael and Daum Robert, 2010). Nevertheless, even in the absence of PVL, MRSA remains a significant pathogen, and its environmental persistence may contribute to further dissemination. These findings again highlight the need to link resistance patterns to respective habitats for a better understanding and further research in the antimicrobial resistance crisis. The *S. aureus* School strain however, did not show significantly increased resistance to desiccation and X-ray irradiation compared to *S. aureus* Type. While the survival fraction of both tested *S. aureus* strains was low after desiccation for 56 days, there was still recoverable survival fraction. Other studies have also shown that *S. aureus* has a remarkable desiccation tolerance with surviving on dry surfaces for over 1,097 days on plastic, or for 203 days on patients’ blankets (Chaibenjawong and Foster, 2011; Noble, 1962). Despite the *S. aureus* School genome displaying the highest AMR potential, its resistance to hydrogen peroxide exposure, desiccation, and biofilm formation was not elevated relative to *S. aureus* Type. This result highlights the distinction between detected genotype and observed phenotype. Moreover, despite increased antibiotic resistance, the overall resistance of *S. aureus* School to environmental stressors did not increase.

Interestingly, survival of the *S. aureus* School isolate was lower than survival of *S. aureus* Type after exposure to hydrogen peroxide. However, survival of *S. aureus* Type after exposure to 3% hydrogen peroxide was highest for all tested *Staphylococcus* spp. Hydrogen peroxide has been tested extensively with MIC concentrations ranging from 0.27 mM to 0.66 mM for *S. aureus* and 0.4 mM to 0.55 mM for *S. epidermidis* (Raval et al., 2021). However, in our testing we used 1.5% and 3% hydrogen peroxide correlating to a molarity of 441 mM and 882 mM respectively, and were still able to see survival of *S. aureus* and *S. epidermidis* after 30 min each. However, we did not see survival of *S. haemolyticus* strains after 30 min exposure to 3% hydrogen peroxide, which is in line with it being a lesser-known strain for biofilm formation.

Biofilm formation is a significant issue in medical devices, which could be a concern for astronauts using medical equipment in space (Flores et al., 2024; Khatoon et al., 2018). Besides *S. aureus* also *S. epidermidis* is among the most common pathogens responsible for hospital-acquired infections, typically characterized by biofilm formation (He et al., 2016; Otto, 2008). One study found that the penetration of β-lactam antibiotics was reduced through biofilms of *S. aureus* and *S. epidermidis* which elucidates the importance of studying biofilm formation across isolates from different habitats (Singh et al., 2010). Hence, we decided to test *S. epidermidis* School as another skin commensal and opportunistic pathogen. *S. epidermidis* is a gram-positive, coagulase-negative *Staphylococcus* species that commonly colonizes human skin and mucous membranes and can act as an opportunistic pathogen, particularly in biofilm-associated infections (Otto, 2009). *S. epidermidis* School only showed resistance to tetracycline, indicating low resistance in this community isolate. A previous study also found that clinical isolates were more resistant to antibiotics compared to community isolates (Michelim et al., 2005). *S. epidermidis* School did not show increased resistance to desiccation, X-ray irradiation, or hydrogen peroxide exposure. However, his biofilm formation potential was significantly increased compared to *S. epidermidis* Type, which is worrying due to its nature of causing biofilm- associated infections. One study showed that treatment of *S. epidermidis* with sub-lethal concentration of hydrogen peroxide led to decreased biofilm formation indicating potential for use in clinical settings (Glynn et al., 2009).

Biofilm formation can be considered an adaptive protective mechanism in response to environmental stressors, including hydrogen peroxide exposure, desiccation, X-ray irradiation, and antibiotic treatments (Henriksen et al., 2022; Muratov et al., 2025; Singh et al., 2010; Ito and Hiramatsu, 1998; Parker et al., 2023; Dengler Haunreiter et al., 2019; Severn and Horswill, 2023). However, in our study we only found elevated biofilm formation in *S. epidermidis* School, but not in *S. aureus* School or in *S. haemolyticus* ISS strains compared to the respective type strain. While increased biofilm formation of *S. epidermidis* School may be of critical concern, the remaining findings suggest that environmental stressors on the ISS and in schools do not exert a greater impact on the isolated strains. This is also in line with our other findings, since we did not find statistically significant differences in the survival of *Staphylococcus* spp. after exposure to desiccation, X-ray irradiation, and hydrogen peroxide.

However, we identified clear differences during testing of *M. luteus* strains. In addition to clinically relevant *Staphylococcus* strains, we also included *M. luteus*, a common skin commensal frequently found in soil, dust, and water. *M. luteus* is a gram-positive, obligate aerobe member of the *Micrococcaceae* family, and generally non-pathogenic but capable of opportunistic infections in immunocompromised individuals (Shi et al., 2023). *M. luteus* has also been regularly found on the ISS before (Mora et al., 2016b; Castro et al., 2004), and has been isolated from 125 million year old amber (Greenblatt et al., 2004) highlighting its potential to enter a prolonged dormancy state and tolerance to diverse stressors. All *M. luteus* strains exhibited a high tolerance to both desiccation and X-ray irradiation. Desiccation tolerance of *Micrococcus* has been hypothesized to be due to intrinsic desiccation resistance (Mora et al., 2016b). Survival of *M. luteus* School 2 and *M. luteus* School 3 even exceeded the initial cell numbers after desiccation until day 56 and day 21, respectively. Desiccation and rehydration could have disrupted cellular aggregates, thereby increasing countable CFU. Remarkably, *M. luteus* is able to resuscitate and stimulate the activity of viable but non-culturable (VBNC) or previously uncultured bacteria through the secretion of a protein called resuscitation-promoting factor (Rpf) (Mukamolova et al., 2006), of which the respective gene was present in all *M. luteus* isolates. Some cells, which had entered the VBNC state, might have been more efficiently resuscitated after desiccation and subsequent rehydration in rich medium, thereby increasing countable CFU. Additionally, entering a VBNC state by *M. luteus* can be indicated by the loss of CFU (Tirumalai et al., 2025). *M. luteus* Type might have shown delayed onset of dormancy under desiccation possibly due to not being pre-adapted to desiccation compared to cleanroom isolates (Tirumalai et al., 2025). In our study, all *M. luteus* isolates from school and ISS were pre-adapted to desiccation due to them being isolated from surfaces after a desiccation period of approximately 6 months (Krämer et al., 2025). Among the tested *M. luteus* strains in this study, *M. luteus* Type showed the highest decrease in survival (~1-log) followed by *M. luteus* ISS. While lower survival of *M. luteus* Type might be linked to no pre-adaptation to desiccation, this is not true for the ISS strain. However, desiccation conditions might have been different between ISS and school isolates. *M. luteus* has been shown to have a high tolerance to ionizing radiation and to adapt to gamma irradiation through adaptive laboratory evolution (Tang et al., 2025), demonstrating its ability to adapt to ionizing irradiation. However, we did not find a statistically significant difference in X-ray irradiation tolerance, which was high in all *M. luteus* strains and survival of *M. luteus* ISS did not decrease less than 2-log reduction. Maybe a pre-adaptation to increased irradiation led to less countable colonies, which indicates an earlier onset of dormancy. Following exposure to 1.5% hydrogen peroxide for up to 30 min, survival of *M. luteus* isolates showed only minor reductions with a maximum decrease of 2-log units. No survival was detected for all *M. luteus* isolate strains after treatment with 3% hydrogen peroxide for 30 min, while *M. luteus* Type showed less than 1-log reduction in survival after 30 min treatment with 3% hydrogen peroxide. An earlier study showed that hydrogen peroxide exposure above 40 mM induced protein damage in *M. luteus* (Lorin-Latxague and Melin, 2005). *M. luteus* ISS exhibited slightly increased MICs towards piperacillin/tazobactam and cefotaxime compared to the other *M. luteus* strain, possibly indicating increased antibiotic resistance. However, the determined MIC values were close to the established breakpoint values (EUCAST, 2024), which may suggest no strong evidence for highly increased resistance in the spaceflight isolate compared to the other isolates. Other studies found that ISS isolates can show increased antibiotic resistance and virulence (Sengupta et al., 2024; Singh et al., 2018). Interestingly, early-stage biofilm formation of ISS strain *M. luteus* ISS was very significantly increased and showed overall the highest biofilm formation. Biofilm formation of *M. luteus* School 1 and *M. luteus* School 2 was also significantly increased compared to *M. luteus* Type. This is in line with findings showing that biofilm formation was enhanced in strains from the ISS (Mora et al., 2019). Another study also found that production of exopolymeric substances (EPS) was higher in the ISS strains of *M. luteus* than in a terrestrial reference strain. Additionally, they found that both ISS as well as the reference strain were growing faster under simulated microgravity than under Earth gravity (Mauclaire and Egli, 2010). However, only early-stage biofilm formation and surface attachment were analyze in this study, assessing biofilm formation kinetics over a prolonged period could aid in gaining a better understanding, as SEM images did not yet reveal a mature biofilm matrix.

The confined and controlled environment of the ISS may promote the selection of antibiotic-resistant bacteria. In particular, confinement has been associated with reduced biodiversity, which may increase the risk of resistance development (Mahnert et al., 2019). In our study, we did not see statistically significant increase in resistance to desiccation, X-ray irradiation or hydrogen peroxide tolerance in ISS and school isolates compared to the respective type strains. Studies have shown that the effectiveness of antibiotics may differ in microgravity, and bacteria might become more resistant or less susceptible to the drugs used on Earth [as reviewed in Taylor (2015)]. With longer missions, such as those planned for Moon or Mars, bacterial resistance could pose a significant challenge for maintaining astronaut health. The isolation, limited medical resources, and confined space of such missions make managing infections much more difficult and while further research is needed regarding the management and treatment of bacterial infections in extraterrestrial habitats [as reviewed in Boschert et al. (2025)], our data does not show a clear trend towards an increased antibiotic resistance of space isolates compared to isolates from terrestrial habitats.

While the ISS provides a unique environment to study the acquisition of bacterial resistance under conditions not replicable on Earth, such as the interplay of microgravity, radiation, and limited resources, our data did not indicate that space-derived isolates were particularly resistant. Other studies have shown some strains isolated from the ISS, such as *Enterobacter bugandensis*, exhibited enhanced antibiotic resistance and increased virulence (Singh et al., 2018; Sengupta et al., 2024). Moreover, *E. bugandensis* strains from the ISS showed a distinctly different genotype compared to terrestrial isolates (Sengupta et al., 2024). However, the absence of a clear trend toward habitat-specific adaptation in isolates recovered from the “Touching Surfaces” project, may be attributed to the short duration of exposure to space conditions, which may have been insufficient to induce measurable adaptation. Limitations related to sample size and exposure duration may be addressed by conducting analogue exposure experiments with systematic monitoring of adaptation levels. However, another study has shown that already short-term cultivation on board the ISS led to increased mutation rates in *S. epidermidis* (Fajardo-cavazos and Nicholson, 2016). To assess habitat effects, future research should include sampling across multiple independent environments and timepoints to distinguish environmental influences from strain-specific variability. Additionally, laboratory evolution experiments could be used to study how for example simulated microgravity impacts adaptation. Moreover, for a more reliable conclusion, more isolates could be tested regarding the effects of space exposure compared to terrestrial settings. However, astronauts’ microbiomes have been shown to change during space missions, with some studies showing shifts in the bacterial communities on the skin, mouth, and gut but also in viral reactivation (Urbaniak et al., 2020; Voorhies et al., 2019). These findings could imply that microbial adaptation can occur within a shorter time-frame, but was either not evident in our isolates or the extraterrestrial stressors may be less severe on the bacteria compared to for example confinement. Studies have shown that especially bacteria which are very well adapted can survive on the ISS and other confined habitats such as intensive care units.

## 5 Conclusion and outlook

We found that the isolates of *Staphylococcus* spp. and *M. luteus* isolates from schools and the ISS did not show an overall increase in antibiotic resistance, desiccation tolerance, X-ray irradiation resistance or resistance to hydrogen peroxide. However, we did see some significant increases in biofilm formation from *S. epidermidis* School, which is important due to its potential to cause biofilm-associated infections. Additionally, we found that the biofilm formation of all school isolates and ISS isolates was increased, and even significantly for most of the strains, which is in line with earlier studies seeing biofilm formation as a threat to the material integrity of spacecrafts (Mora et al., 2019). Overall, our results do not show strong evidence that ISS isolates are more resistant than school isolates under the tested conditions.

A well-structured surveillance system is needed to monitor microbial resistance especially in confined habitats such as space habitats, which will also contribute to public health data on Earth. While sequencing has brought great advancement to the field and has enabled more in-depth analysis of the indoor microbiome, cultivation is essential for investigating resistance potential of detected strains, and linking of microbial genotypes to phenotypes. This also is true for investigation of viruses and filamentous fungi in space habitats, to ensure a broad overview and assess the real risk of microbial presence in space habitats. Studying resistance of bacteria from space and thereby contributing to the understanding of resistance and co-resistance may lead to the development of new technologies or treatments to combat antibiotic-resistant bacteria not only in space but most importantly on Earth.

## Data Availability

The datasets presented in this study can be found in online repositories. The names of the repository/repositories and accession number(s) can be found at https://www.ncbi.nlm.nih.gov/genbank/, PRJNA1445116.
