# Supplementary material for: Bacterial resistance across habitats: from German schools to the International Space Station
Source: Front Microbiol. 2026 Jun 18;17:1849378. doi: 10.3389/fmicb.2026.1849378 (PMC13323154; doi:10.3389/fmicb.2026.1849378)
Supplement: Supplementary file 1 [file Data_Sheet_1.DOCX]

Supplementary Material

# Supplementary Data

## Supplementary Figures


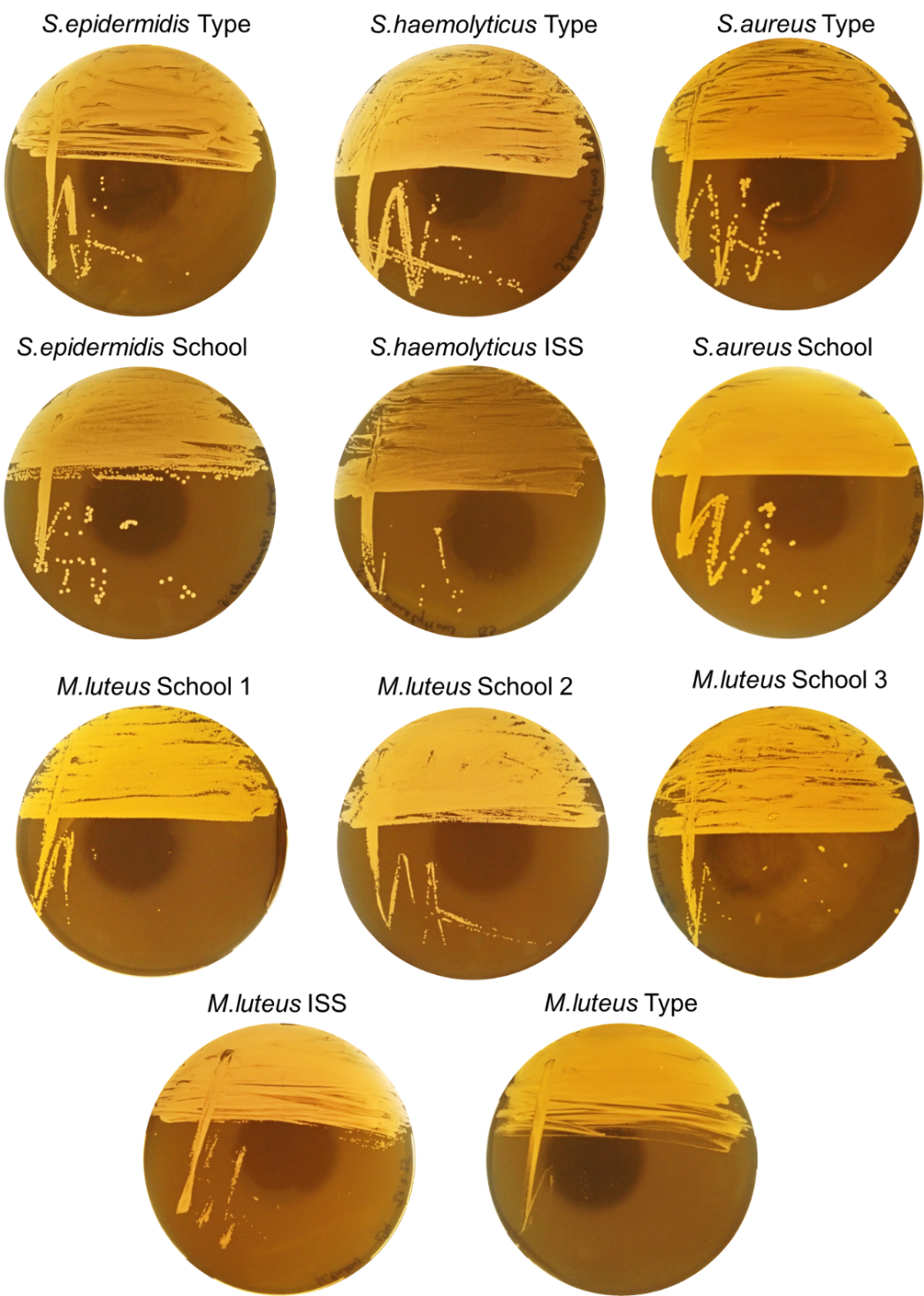


Supplementary Figure 1: Overview of *Staphylococcus* and *M. luteus* isolates. Isolates were grown on TSYA plates and incubated overnight at 37°C.

## Supplementary Tables

Supplementary Table 1: Setup of microbroth dilution assay for determination of minimal inhibitory concentration in 96-well plate format.

|  | **1** | **2** | **3** | **4** | **5** | **6** | **7** | **8** | **9** | **10** | **11** | **12** |
| --- | --- | --- | --- | --- | --- | --- | --- | --- | --- | --- | --- | --- |
| **A** | Penicillin 8 µg/mL | Ampicillin 16 µg/mL | Oxacillin 16 µg/mL | Rifampicin 2 µg/mL | Teicoplanin 16 µg/mL | Vancomycin 32 µg/mL | Fosfomycin 64 µg/mL | Linezolid 8 µg/mL | Tigecyline 1 µg/mL | Gentamycin high level 128 µg/mL | Gentamycin high level 500 µg/mL | Mupirocin 256 µg/mL |
| **B** | Penicillin 4 µg/mL | Ampicillin 8 µg/mL | Oxacillin 8 µg/mL | Rifampicin 1 µg/mL | Teicoplanin 8 µg/mL | Vancomycin 16 µg/mL | Fosfomycin 32 µg/mL | Linezolid 4 µg/mL | Tigecyline 0.5 µg/mL | Trimethoprim/Sulfamethoxazole 4/76 µg/mL | Fusidic acid 2 µg/mL | Mupirocin 1 µg/mL |
| **C** | Penicillin 2 µg/mL | Ampicillin 4 µg/mL | Oxacillin 4 µg/mL | Rifampicin 0.5 µg/mL | Teicoplanin 4 µg/mL | Vancomycin 8 µg/mL | Fosfomycin 16 µg/mL | Linezolid 2 µg/mL | Tigecyline 0.25 µg/mL | Trimethoprim/Sulfamethoxazole 2/38 µg/mL | Fusidic acid 1 µg/mL | Erythromycin 4 µg/mL |
| **D** | Penicillin 1 µg/mL | Ampicillin 2 µg/mL | Oxacillin 2 µg/mL | Rifampicin 0.0625 µg/mL | Teicoplanin 2 µg/mL | Vancomycin 4 µg/mL | Fosfomycin 8 µg/mL | Linezolid 1 µg/mL | Tigecyline 0.125 µg/mL | Trimethoprim/Sulfamethoxazole 1/19 µg/mL | Gentamycin 8 µg/mL | Erythromycin 2 µg/mL |
| **E** | Penicillin 0.5 µg/mL | Ceftarolin 2 µg/mL | Oxacillin 16 µg/mL | Cefoxitin 16 µg/mL | Teicoplanin 1 µg/mL | Vancomycin 2 µg/mL | Quinupristin/Dalfopristin (Synercid) 4 µg/mL | Daptomycin 4 µg/mL | Moxifloxacin 2 µg/mL | Trimethoprim/Sulfamethoxazole 0.5/9.5 µg/mL | Gentamycin 4 µg/mL | Erythromycin 1 µg/mL |
| **F** | Penicillin 0.25 µg/mL | Ceftarolin 1 µg/mL | Oxacillin 0.5 µg/mL | Cefoxitin 8 µg/mL | Teicoplanin 0.5 µg/mL | Vancomycin 1 µg/mL | Quinupristin/Dalfopristin (Synercid) µg/mL | Daptomycin 2 µg/mL | Moxifloxacin 1 µg/mL | Trimethoprim/Sulfamethoxazole 0.03125/ 0.59375 µg/mL | Gentamycin 2 µg/mL | Erythromycin 0.5 µg/mL |
| **G** | Penicillin 0.125 µg/mL | Ceftarolin 0.5 µg/mL | Oxacillin 0.25 µg/mL | Cefoxitin 4 µg/mL | Teicoplanin 0.25 µg/mL | Vancomycin 0.5 µg/mL | Quinupristin/Dalfopristin (Synercid) 1 µg/mL | Daptomycin 1 µg/mL | Moxifloxacin 0.5 µg/mL | Erythromycin/Clindamycin 4/0.5 µg/mL | Gentamycin 1 µg/mL | Erythromycin 0.25 µg/mL |
| **H** | Penicillin 0.0625 µg/mL | Ceftarolin 0.25 µg/mL | Oxacillin 0.125 µg/mL | Cefoxitin 2 µg/mL | Teicoplanin 0.125 µg/mL | Vancomycin 0.25 µg/mL | Quinupristin/Dalfopristin (Synercid) 0.5 µg/mL | Daptomycin 0.5 µg/mL | Moxifloxacin 0.25 µg/mL | Clindamycin 0.5 µg/mL | Gentamycin 0.5 µg/mL | Growth Control |

Supplementary Table 2: Used MIC (minimal inhibitory concentration) test strips and their respective manufacturer.

| Used MIC test strips | Manufacturer |
| --- | --- |
| Meropenem high MIC Test Strip 0.016 - 256µg/mL (MRP) | *Liofilchem, Italy* |
| Ciprofloxacin MIC Test Strip 0.002 - 32µg/mL (CIP) | *Liofilchem, Italy* |
| Linezolid MIC Test Strip 0.016 - 256 µg/mL (LNZ) | *Liofilchem, Italy* |
| Penicillin G high MIC Test Strip 0.016 - 256µg/mL (P) | *Liofilchem, Italy* |
| Ampicillin MIC Test Strip 0.016 - 256 µg/mL (AMP) | *Liofilchem, Italy* |
| Cefotaxim high MIC Test Strip 0.016 - 256 µg/mL (CTX) | *Liofilchem, Italy* |
| Ceftazidim MIC Test Strip 0.016 - 256 µg/mL (CAZ) | *Liofilchem, Italy* |
| Cefepim high MIC Test Strip 0.016 - 256µg/mL (FEP) | *Liofilchem, Italy* |
| Imipenem high MIC Test Strip 0.016 - 256 µg/mL (IMI) | *Liofilchem, Italy* |
| Clindamycin MIC Test Strip 0.016 - 256µg/mL (CD) | *Liofilchem, Italy* |
| Linezolid MIC Test Strip 0.016 - 256 µg/mL (LNZ) | *Liofilchem, Italy* |
| Vancomycin MIC Test Strip 0.016 - 256µg/mL (VA) | *Liofilchem, Italy* |
| Piperacillin/Tazobactam P/T 0,016-256 µg/mL (P/T) | *bioMerieux, France* |

Supplementary Table 3: Antimicrobial resistance genes of *Staphylococcus* spp. retrieved using ABRicate (megares database).

| **Strain** | **Start** | **End** | **Strand** | **Gene** | **Coverage** | **Coverage map** | **Gaps** | **% coverage** | **% identity** | **Accession** | **Product** |
| --- | --- | --- | --- | --- | --- | --- | --- | --- | --- | --- | --- |
| *S. aureus* Type | 33169 | 33644 | + | *rlmH* | 1-476/480 | =============== | 0/0 | 99.17 | 99.58 | MEG_6058 | Drugs:MLS:23S_rRNA_methyltransferases:rlmH |
|  | 101731 | 103083 | + | *tet(38)* | 1-1353/1353 | =============== | 0/0 | 100 | 100 | MEG_6982 | Drugs:Tetracyclines:Tetracycline_resistance_MFS_efflux_pumps:tet(38) |
|  | 332013 | 332432 | + | *mepR* | 1-420/420 | =============== | 0/0 | 100 | 99.29 | MEG_3836 | Multi-compound:Drug_and_biocide_resistance:Drug_and_biocide_MATE_efflux_regulator:MepR |
|  | 332524 | 333894 | + | *mepA* | 1-1371/1371 | =============== | 0/0 | 100 | 100 | MEG_3834 | Multi-compound:Drug_and_biocide_resistance:Drug_and_biocide_MATE_efflux_pumps:mepA |
|  | 333998 | 334438 | + | *mepB* | 1-441/441 | =============== | 0/0 | 100 | 100 | MEG_3835 | Drugs:Multi-drug_resistance:Multi-drug_MATE_efflux_pump:mepB |
|  | 689213 | 689656 | + | *aac(3)* | 1-444/444 | =============== | 0/0 | 100 | 98.87 | MEG_47 | Drugs:Aminoglycosides:Aminoglycoside_N-acetyltransferases:aac(3) |
|  | 697679 | 698122 | - | *mgrA* | 1-444/444 | =============== | 0/0 | 100 | 99.78 | MEG_3943 | Drugs:Multi-drug_resistance:MDR_regulator:MgrA |
|  | 705772 | 706938 | + | *norA* | 1-1167/1167 | =============== | 0/0 | 100 | 99.91 | MEG_4207 | Multi-compound:Drug_and_biocide_resistance:Drug_and_biocide_MFS_efflux_pumps:norA |
|  | 1421816 | 1423171 | - | *arlS* | 1-1356/1356 | =============== | 0/0 | 100 | 100 | MEG_1118 | Drugs:Multi-drug_resistance:MDR_regulator:ArlS |
|  | 1423168 | 1423827 | - | *arlR* | 1-660/660 | =============== | 0/0 | 100 | 100 | MEG_1117 | Drugs:Multi-drug_resistance:MDR_regulator:ArlR |
|  | 1469899 | 1471290 | - | *norB* | 1-1392/1392 | =============== | 0/0 | 100 | 99.28 | MEG_4210 | Multi-compound:Drug_and_biocide_resistance:Drug_and_biocide_MFS_efflux_pumps:norB |
|  | 1790739 | 1791539 | - | *aph(3')-IIa* | 1-801/801 | =============== | 0/0 | 100 | 100 | MEG_1060 | Drugs:Aminoglycosides:Aminoglycoside_O-phosphotransferases:APH3-PRIME |
|  | 2180121 | 2181563 | - | *lmrS* | 1-1443/1443 | =============== | 0/0 | 100 | 100 | MEG_3599 | Multi-compound:Drug_and_biocide_resistance:Drug_and_biocide_MFS_efflux_pumps:lmrS |
|  | 2333498 | 2333917 | + | *fosB* | 1-420/420 | =============== | 0/0 | 100 | 100 | MEG_3009 | Drugs:Fosfomycin:Fosfomycin_thiol_transferases:fosB |
|  | 2463307 | 2464493 | - | *dhaP* | 2-1188/1188 | =============== | 0/0 | 99.92 | 99.41 | MEG_2614 | Drugs:Phenicol:Phenicol_resistance_MFS_efflux_pumps:DHAP |
| *S. aureus* School | 33162 | 33639 | + | *rlmH* | 1-478/480 | =============== | 0/0 | 99.58 | 98.54 | MEG_6058 | Drugs:MLS:23S_rRNA_methyltransferases:rlmH |
|  | 41766 | 43775 | - | *mecA* | 1-2010/2010 | =============== | 0/0 | 100 | 99.95 | MEG_3785 | Drugs:betalactams:Penicillin_binding_protein:mecA |
|  | 119459 | 120811 | + | *tet(38)* | 1-1353/1353 | =============== | 0/0 | 100 | 100 | MEG_6982 | Drugs:Tetracyclines:Tetracycline_resistance_MFS_efflux_pumps:tet(38) |
|  | 351916 | 352335 | + | *mepR* | 1-420/420 | =============== | 0/0 | 100 | 99.29 | MEG_3836 | Multi-compound:Drug_and_biocide_resistance:Drug_and_biocide_MATE_efflux_regulator:MepR |
|  | 352427 | 353797 | + | *mepA* | 1-1371/1371 | =============== | 0/0 | 100 | 99.71 | MEG_3834 | Multi-compound:Drug_and_biocide_resistance:Drug_and_biocide_MATE_efflux_pumps:mepA |
|  | 353900 | 354340 | + | *mepB* | 1-441/441 | =============== | 0/0 | 100 | 89.34 | MEG_3835 | Drugs:Multi-drug_resistance:Multi-drug_MATE_efflux_pump:mepB |
|  | 705755 | 706198 | + | *aac(3)* | 1-444/444 | =============== | 0/0 | 100 | 99.1 | MEG_47 | Drugs:Aminoglycosides:Aminoglycoside_N-acetyltransferases:aac(3) |
|  | 714221 | 714664 | - | *mgrA* | 1-444/444 | =============== | 0/0 | 100 | 100 | MEG_3943 | Drugs:Multi-drug_resistance:MDR_regulator:MgrA |
|  | 722315 | 723481 | + | *norA* | 1-1167/1167 | =============== | 0/0 | 100 | 99.91 | MEG_4207 | Multi-compound:Drug_and_biocide_resistance:Drug_and_biocide_MFS_efflux_pumps:norA |
|  | 1407004 | 1408359 | - | *arlS* | 1-1356/1356 | =============== | 0/0 | 100 | 99.11 | MEG_1118 | Drugs:Multi-drug_resistance:MDR_regulator:ArlS |
|  | 1408356 | 1409015 | - | *arlR* | 1-660/660 | =============== | 0/0 | 100 | 99.55 | MEG_1117 | Drugs:Multi-drug_resistance:MDR_regulator:ArlR |
|  | 1454348 | 1455739 | - | *norB* | 1-1392/1392 | =============== | 0/0 | 100 | 98.56 | MEG_4210 | Multi-compound:Drug_and_biocide_resistance:Drug_and_biocide_MFS_efflux_pumps:norB |
|  | 1776430 | 1777230 | - | *aph(3')-IIa* | 1-801/801 | =============== | 0/0 | 100 | 99.75 | MEG_1060 | Drugs:Aminoglycosides:Aminoglycoside_O-phosphotransferases:APH3-PRIME |
|  | 2187704 | 2189146 | - | *lmrS* | 1-1443/1443 | =============== | 0/0 | 100 | 99.93 | MEG_3599 | Multi-compound:Drug_and_biocide_resistance:Drug_and_biocide_MFS_efflux_pumps:lmrS |
|  | 2464746 | 2465933 | - | *dhaP* | 1-1188/1188 | =============== | 0/0 | 100 | 99.66 | MEG_2614 | Drugs:Phenicol:Phenicol_resistance_MFS_efflux_pumps:DHAP |
|  | 10003 | 11478 | + | *aac(6')-I* | 1-1476/1476 | =============== | 0/0 | 100 | 100 | MEG_352 | Drugs:Aminoglycosides:Aminoglycoside_N-acetyltransferases:AAC6-PRIME |
|  | 12034 | 12796 | + | *ermB* | 1-762/762 | ========/====== | 1.00 | 100 | 99.21 | MEG_2801 | Drugs:MLS:23S_rRNA_methyltransferases:ermB |
|  | 25687 | 26067 | - | *blaI* | 1-381/381 | =============== | 0/0 | 100 | 98.42 | MEG_1287 | Drugs:betalactams:Penicillin_binding_protein_regulator:blaI |
|  | 26057 | 27814 | - | *blaR* | 1-1758/1758 | =============== | 0/0 | 100 | 97.27 | MEG_1299 | Drugs:betalactams:Penicillin_binding_protein_regulator:blaR |
|  | 27879 | 28766 | + | *blaZ* | 1-888/888 | =============== | 0/0 | 100 | 99.21 | MEG_1409 | Drugs:betalactams:Class_A_betalactamases:blaZ |
| *S. epidermidis* Type | 211628 | 212056 | + | *fosB* | 1-429/429 | =============== | 0/0 | 100 | 99.77 | MEG_3007 | Drugs:Fosfomycin:Fosfomycin_thiol_transferases:fosB |
|  | 931991 | 932888 | - | *blaZ* | 3-900/900 | =============== | 0/0 | 99.78 | 99 | MEG_1413 | Drugs:betalactams:Class_A_betalactamases:blaZ |
|  | 932943 | 934700 | + | *blaR* | 1-1758/1758 | =============== | 0/0 | 100 | 99.49 | MEG_1299 | Drugs:betalactams:Penicillin_binding_protein_regulator:blaR |
|  | 934690 | 935070 | + | *blaI* | 1-381/381 | =============== | 0/0 | 100 | 98.95 | MEG_1287 | Drugs:betalactams:Penicillin_binding_protein_regulator:blaI |
|  | 1095152 | 1095952 | + | *aph(3')-IIa* | 1-801/801 | =============== | 0/0 | 100 | 83.15 | MEG_1060 | Drugs:Aminoglycosides:Aminoglycoside_O-phosphotransferases:APH3-PRIME |
|  | 2087806 | 2088969 | - | *norA* | 1-1164/1164 | =============== | 0/0 | 100 | 100 | MEG_4208 | Multi-compound:Drug_and_biocide_resistance:Drug_and_biocide_MFS_efflux_pumps:norA |
|  | 2096104 | 2096547 | + | *mgrA* | 1-444/444 | =============== | 0/0 | 100 | 88.51 | MEG_3943 | Drugs:Multi-drug_resistance:MDR_regulator:MgrA |
| *S. epidermidis* School | 31693 | 32172 | + | *rlmH* | 1-480/480 | =============== | 0/0 | 100 | 81.46 | MEG_6058 | Drugs:MLS:23S_rRNA_methyltransferases:rlmH |
|  | 162730 | 163158 | + | *fosB* | 1-429/429 | =============== | 0/0 | 100 | 99.77 | MEG_3007 | Drugs:Fosfomycin:Fosfomycin_thiol_transferases:fosB |
|  | 1033686 | 1034486 | + | *aph(3')-IIa* | 1-801/801 | =============== | 0/0 | 100 | 83.15 | MEG_1060 | Drugs:Aminoglycosides:Aminoglycoside_O-phosphotransferases:APH3-PRIME |
|  | 2045658 | 2046821 | - | *norA* | 1-1164/1164 | =============== | 0/0 | 100 | 99.91 | MEG_4208 | Multi-compound:Drug_and_biocide_resistance:Drug_and_biocide_MFS_efflux_pumps:norA |
|  | 2053956 | 2054399 | + | *mgrA* | 1-444/444 | =============== | 0/0 | 100 | 88.51 | MEG_3943 | Drugs:Multi-drug_resistance:MDR_regulator:MgrA |
| *S. haemolyticus* Type | 28796 | 29273 | + | *rlmH* | 1-478/480 | =============== | 0/0 | 99.58 | 82.64 | MEG_6058 | Drugs:MLS:23S_rRNA_methyltransferases:rlmH |
|  | 1117307 | 1118107 | + | *aph(3')-IIa* | 1-801/801 | ========/====== | 2 | 99.88 | 82.79 | MEG_1060 | Drugs:Aminoglycosides:Aminoglycoside_O-phosphotransferases:APH3-PRIME |
|  | 2119226 | 2119665 | + | *mgrA* | 1-440/444 | =============== | 0/0 | 99.1 | 81.82 | MEG_3943 | Drugs:Multi-drug_resistance:MDR_regulator:MgrA |
| *S. haemolyticus* ISS | 28795 | 29272 | + | *rlmH* | 1-478/480 | =============== | 0/0 | 99.58 | 82.43 | MEG_6058 | Drugs:MLS:23S_rRNA_methyltransferases:rlmH |
|  | 1101010 | 1101810 | + | *aph(3')-IIa* | 1-801/801 | ========/====== | 2 | 99.88 | 82.79 | MEG_1060 | Drugs:Aminoglycosides:Aminoglycoside_O-phosphotransferases:APH3-PRIME |
|  | 2103716 | 2104155 | + | *mgrA* | 1-440/444 | =============== | 0/0 | 99.1 | 81.82 | MEG_3943 | Drugs:Multi-drug_resistance:MDR_regulator:MgrA |
|  | 19923 | 21389 | + | *msrA* | 1-1467/1467 | =============== | 0/0 | 100 | 99.93 | MEG_4066 | Drugs:MLS:MLS_resistance_ABC_efflux_pumps:MSRA |
|  | 21488 | 22387 | + | *mphC* | 1-900/900 | =============== | 0/0 | 100 | 100 | MEG_4038 | Drugs:MLS:Macrolide_phosphotransferases:MPHC |
|  | 23686 | 24252 | - | *qacR* | 1-567/567 | =============== | 0/0 | 100 | 99.82 | MEG_5856 | Multi-compound:Drug_and_biocide_resistance:Drug_and_biocide_MFS_efflux_regulator:QACR |
|  | 24430 | 25974 | + | *qacAB* | 1-1545/1545 | =============== | 0/0 | 100 | 99.94 | MEG_5823 | Multi-compound:Drug_and_biocide_resistance:Drug_and_biocide_MFS_efflux_pumps:QACAB |

Supplementary Table 4: Antimicrobial resistance genes of *M. luteus* strains retrieved using ABRicate (megares).

| **Strain** | **Start** | **End** | **Strand** | **Gene** | **Coverage** | **Coverage map** | **Gaps** | **% coverage** | **% identity** | **Accession** | **Product** |
| --- | --- | --- | --- | --- | --- | --- | --- | --- | --- | --- | --- |
| *M. luteus* Type | 1563 | 2357 | - | *aph(3')-IIa* | 1-795/795 | =============== | 0/0 | 100 | 100 | MEG_1050 | Drugs:Aminoglycosides:Aminoglycoside_O-phosphotransferases:APH3-DPRIME |
|  | 5301 | 6241 | + | *blaTEM* | 73-1013/1091 | .=============. | 0/0 | 86.25 | 99.68 | MEG_6879 | Drugs:betalactams:Class_A_betalactamases:TEM |
| *M. luteus* School 1 | 856 | 1989 | + | *blaTEM* | 175-1308/1391 | .============== | 0/0 | 81.52 | 99.38 | MEG_6909 | Drugs:betalactams:Class_A_betalactamases:TEM |
| *M. luteus* School 2 | 4626 | 5759 | + | *blaTEM* | 175-1308/1391 | .============== | 0/0 | 81.52 | 99.38 | MEG_6909 | Drugs:betalactams:Class_A_betalactamases:TEM |
|  | 6532 | 7326 | - | *aph(3')-IIa* | 1-795/795 | =============== | 0/0 | 100 | 100 | MEG_1050 | Drugs:Aminoglycosides:Aminoglycoside_O-phosphotransferases:APH3-DPRIME |
| *M. luteus* School 3 | 2999 | 4134 | + | blaTEM | 173-1308/1391 | .============== | 0/0 | 81.67 | 99.38 | MEG_6909 | Drugs:betalactams:Class_A_betalactamases:TEM |
|  | 5502 | 6287 | - | aph(3')-IIa | 10-795/795 | =============== | 0/0 | 98.87 | 100 | MEG_1050 | Drugs:Aminoglycosides:Aminoglycoside_O-phosphotransferases:APH3-DPRIME |
| *M. luteus* ISS | 6242 | 7375 | + | blaTEM | 175-1308/1391 | .============== | 0/0 | 81.52 | 99.38 | MEG_6909 | Drugs:betalactams:Class_A_betalactamases:TEM |

Supplementary Table 5: Presence or absence of detected antimicrobial and virulence related genes of *S. aureus* strains using ABRicate with different databases and the respective identity coverage.

| Strain | *S. aureus* Type | | | | *S. aureus* School | | | |
| --- | --- | --- | --- | --- | --- | --- | --- | --- |
| Database | megares | ncbi | resfinder | vfdb | megares | ncbi | resfinder | vfdb |
| number of genes found | 15 | 2 | 0 | 61 | 20 | 7 | 4 | 67 |
| *aac(3)* | 100 | . | . | . | 100 | . | . | . |
| *aac(6‘)-I* | . | . | . | . | 100 | . | . | . |
| *adsA* | . | . | . | 100 | . | . | . | 100 |
| *aph(2'')-Ia_2* | . | . | . | . | . | . | 100 | . |
| *aph(2'')-Ih* | . | . | . | . | . | 89.15 | . | . |
| *aph(3‘)-I* | 100 | . | . | . | 100 | . | . | . |
| *arlR* | 100 | . | . | . | 100 | . | . | . |
| *arlS* | 100 | . | . | . | 100 | . | . | . |
| *aur* | . | . | . | 100 | . | . | . | 100 |
| *blaI* | . | . | . | . | 100 | . | . | . |
| *blaI_of_Z* | . | . | . | . | . | 100 | . | . |
| *blaR* | . | . | . | . | 100 | . | . | . |
| *blaR1* | . | . | . | . | . | 100 | . | . |
| *blaTEM-116* | . | . | . | . | . | . | . | . |
| *blaZ* | . | . | . | . | 100 | 100 | . | . |
| *blaZ_79* | . | . | . | . | . | . | 100 | . |
| *cap8A* | . | . | . | 100 | . | . | . | 100 |
| *cap8B* | . | . | . | 100 | . | . | . | 100 |
| *cap8C* | . | . | . | 100 | . | . | . | 100 |
| *cap8D* | . | . | . | 100 | . | . | . | 100 |
| *cap8E* | . | . | . | 100 | . | . | . | 100 |
| *cap8F* | . | . | . | 100 | . | . | . | 100 |
| *cap8G* | . | . | . | 100 | . | . | . | 100 |
| *cap8H* | . | . | . | . | . | . | . | 100 |
| *cap8I* | . | . | . | . | . | . | . | 100 |
| *cap8J* | . | . | . | . | . | . | . | 100 |
| *cap8K* | . | . | . | . | . | . | . | 100 |
| *cap8L* | . | . | . | 100 | . | . | . | 100 |
| *cap8M* | . | . | . | 100 | . | . | . | 100 |
| *cap8N* | . | . | . | 100 | . | . | . | 100 |
| *cap8O* | . | . | . | 100 | . | . | . | 100 |
| *cap8P* | . | . | . | 100 | . | . | . | 100 |
| *chp* | . | . | . | . | . | . | . | 100 |
| *clfA* | . | . | . | 95.99 | . | . | . | 99.3 |
| *clfB* | . | . | . | 96.59 | . | . | . | 96.81 |
| *coa* | . | . | . | 98.58 | . | . | . | . |
| *dhaP* | 99.92 | . | . | . | 100 | . | . | . |
| *ebp* | . | . | . | 100 | . | . | . | 100 |
| *erm(B)* | . | . | . | . | 100 | 100 | 100 | . |
| *esaA* | . | . | . | 100 | . | . | . | 100 |
| *esaB* | . | . | . | 100 | . | . | . | 100 |
| *esaC* | . | . | . | 100 | . | . | . | 100 |
| *essA* | . | . | . | 100 | . | . | . | 100 |
| *essB* | . | . | . | 100 | . | . | . | 100 |
| *essC* | . | . | . | 100 | . | . | . | 100 |
| *esxA* | . | . | . | 100 | . | . | . | 100 |
| *esxB* | . | . | . | 100 | . | . | . | 100 |
| *fnbA* | . | . | . | 99.64 | . | . | . | 99.64 |
| *fnbB* | . | . | . | 98.91 | . | . | . | . |
| *fosB* | 100 | . | . | . | . | . | . | . |
| *fosB-Saur* | . | 100 | . | . | . | . | . | . |
| *geh* | . | . | . | 100 | . | . | . | 100 |
| *hlb* | . | . | . | 100 | . | . | . | 82.98 |
| *hld* | . | . | . | 100 | . | . | . | 100 |
| *hlgA* | . | . | . | 100 | . | . | . | 100 |
| *hlgB* | . | . | . | 100 | . | . | . | 100 |
| *hlgC* | . | . | . | 100 | . | . | . | 100 |
| *hly/hla* | . | . | . | 100 | . | . | . | 100 |
| *hysA* | . | . | . | 98.53 | . | . | . | 98.73 |
| *icaA* | . | . | . | 100 | . | . | . | 100 |
| *icaB* | . | . | . | 100 | . | . | . | 100 |
| *icaC* | . | . | . | 100 | . | . | . | 100 |
| *icaD* | . | . | . | 100 | . | . | . | 100 |
| *icaR* | . | . | . | 100 | . | . | . | 100 |
| *isdA* | . | . | . | 100 | . | . | . | 100 |
| *isdB* | . | . | . | 100 | . | . | . | 99.69 |
| *isdC* | . | . | . | 100 | . | . | . | 100 |
| *isdD* | . | . | . | 100 | . | . | . | 100 |
| *isdE* | . | . | . | 100 | . | . | . | 100 |
| *isdF* | . | . | . | 100 | . | . | . | 100 |
| *isdG* | . | . | . | 100 | . | . | . | 100 |
| *lip* | . | . | . | 100 | . | . | . | 100 |
| *lmrS* | 100 | . | . | . | 100 | . | . | . |
| *lukF-PV* | . | . | . | 98.98 | . | . | . | 98.98 |
| *map* | . | . | . | 100 | . | . | . | 99.6 |
| *mecA* | . | . | . | . | 100 | 100 | 100 | . |
| *mepA* | 100 | . | . | . | 100 | . | . | . |
| *mepB* | 100 | . | . | . | 100 | . | . | . |
| *mepR* | 100 | . | . | . | 100 | . | . | . |
| *mgrA* | 100 | . | . | . | 100 | . | . | . |
| *norA* | 100 | . | . | . | 100 | . | . | . |
| *norB* | 100 | . | . | . | 100 | . | . | . |
| *rlmH* | 99.17 | . | . | . | 99.58 | . | . | . |
| *sak* | . | . | . | . | . | . | . | 100 |
| *sbi* | . | . | . | 99.77 | . | . | . | 97.11 |
| *scn* | . | . | . | . | . | . | . | 100 |
| *sdrC* | . | . | . | 93.44 | . | . | . | 99.3 |
| *sdrD* | . | . | . | 99.98 | . | . | . | 94.96 |
| *sdrE* | . | . | . | 100 | . | . | . | 91.51 |
| *sec* | . | . | . | . | . | . | . | 100 |
| *sell* | . | . | . | . | . | . | . | 100 |
| *spa* | . | . | . | 100 | . | . | . | . |
| *srtB* | . | . | . | 100 | . | . | . | 100 |
| *sspA* | . | . | . | 100 | . | . | . | 100 |
| *sspB* | . | . | . | 100 | . | . | . | 100 |
| *sspC* | . | . | . | 100 | . | . | . | 100 |
| *tet(38)* | 100 | 100 | . | . | 100 | 100 | . | . |
| *tsst-1* | . | . | . | . | . | . | . | 100 |
| *vWbp* | . | . | . | 99.93 | . | . | . | . |

Supplementary Table 6: Presence or absence of detected antimicrobial and virulence related genes of *S. epidermidis* strains using ABRicate with different databases and the respective identity coverage.

| Strain | *S. epidermidis* Type | | | | *S. epidermidis* School | | | |
| --- | --- | --- | --- | --- | --- | --- | --- | --- |
| Database | megares | ncbi | resfinder | vfdb | megares | ncbi | resfinder | vfdb |
| Number of genes found | 7 | 5 | 2 | 0 | 5 | 2 | 1 | 0 |
| *aph(3')-I* | 100 | . | . | . | 100 | . | . | . |
| *blaI* | 100 | . | . | . | . | . | . | . |
| *blaI_of_Z* | . | 100 | . | . | . | . | . | . |
| *blaR* | 100 | . | . | . | . | . | . | . |
| *blaR1* | . | 100 | . | . | . | . | . | . |
| *blaZ* | 99.78 | 100 | 99.78 | . | . | . | . | . |
| *dfrC* | . | 100 | . | . | . | 100 | . | . |
| *fosB* | 100 | . | . | . | 100 | . | . | . |
| *fosB_4* | . | . | 100 | . | . | . | 100 | . |
| *fosB-251804940* | . | 100 | . | . | . | 100 | . | . |
| *mgrA* | 100 | . | . | . | 100 | . | . | . |
| *norA* | 100 | . | . | . | 100 | . | . | . |
| *rlmH* | . | . | . | . | 100 | . | . | . |

Supplementary Table 7: Presence or absence of detected antimicrobial and virulence related genes of *S. haemolyticus* strains using ABRicate with different databases and the respective identity coverage.

| Strain | *S. haemolyticus* Type | | | | *S. haemolyticus* ISS | | | |
| --- | --- | --- | --- | --- | --- | --- | --- | --- |
| Database | megares | ncbi | resfinder | vfdb | megares | ncbi | resfinder | vfdb |
| number of genes found | 3 | 0 | 0 | 0 | 2 | 7 | 2 | 0 |
| *aph(3')-I* | 99.88 | . | . | . | . | 99.88 | . | . |
| *mgrA* | 99.1 | . | . | . | . | 99.1 | . | . |
| *mphC* | . | . | . | . | 100 | 100 | 100 | . |
| *msrA* | . | . | . | . | 100 | 100 | 100 | . |
| *qacAB* | . | . | . | . | . | 100 | . | . |
| *qacR* | . | . | . | . | . | 100 | . | . |
| *rlmH* | 99.58 | . | . | . | . | 99.58 | . | . |

Supplementary Table 8: Presence or absence of detected antimicrobial and virulence related genes of *M. luteus* strains using ABRicate with different databases and the respective identity coverage.

| **Strain** | **Database** | **Number of genes found** | ***aph(3')*** | ***blaTEM*** |
| --- | --- | --- | --- | --- |
| ***M. luteus* School 1** | megares | 1 | . | 81.52 |
|  | ncbi | 1 | . | 100 |
|  | resfinder | 1 | . | 100 |
|  | vfdb | 0 | . | . |
| ***M. luteus* School 2** | megares | 2 | 100 | 81.52 |
|  | ncbi | 2 | 100 | 100 |
|  | resfinder | 2 | 100 | 100 |
|  | vfdb | 0 | . | . |
| ***M. luteus* School 3** | megares | 2 | 98.87 | 81.67 |
|  | ncbi | 2 | 98.87 | 100 |
|  | resfinder | 2 | 98.87 | 100 |
|  | vfdb | 0 | . | . |
| ***M. luteus* ISS** | megares | 1 | . | 81.52 |
|  | ncbi | 1 | . | 100 |
|  | resfinder | 1 | . | 100 |
|  | vfdb | 0 | . | . |
| ***M. luteus* Type** | megares | 2 | 100 | 86.25 |
|  | ncbi | 2 | 100 | 93.5 |
|  | resfinder | 2 | 100 | 93.5 |
|  | vfdb | 0 | . | . |
